# Neonatal inpatient dataset for small and sick newborn care in low- and middle-income countries: systematic development and multi-country operationalisation with NEST360

**DOI:** 10.1186/s12887-023-04341-2

**Published:** 2023-11-15

**Authors:** James H. Cross, Christine Bohne, Samuel K. Ngwala, Josephine Shabani, John Wainaina, Olabisi Dosunmu, Irabi Kassim, Rebecca E. Penzias, Robert Tillya, David Gathara, Evelyn Zimba, Veronica Chinyere Ezeaka, Opeyemi Odedere, Msandeni Chiume, Nahya Salim, Kondwani Kawaza, Norman Lufesi, Grace Irimu, Olukemi O. Tongo, Lucas Malla, Chris Paton, Louise T. Day, Maria Oden, Rebecca Richards-Kortum, Elizabeth M. Molyneux, Eric O. Ohuma, Joy E. Lawn, with NEST360 Neonatal Inpatient Dataset Learning Group, Aba Asibon, Steve Adudans, Dickson Otiangala, Christina Mchoma, Simeon Yosefe, Adeleke Balogun, Sylvia Omoke, Ekran Rashid, Honorati Masanja, Mike English, Christiane Hagel

**Affiliations:** 1 Maternal, Adolescent, Reproductive, & Child Health Centre, London School of Hygiene & Tropical Medicine, London, UK; 2 Rice360 Institute for Global Health Technologies, Rice University, Texas, USA; 3 Ifakara Health Institute, Ifakara, Tanzania; 4 Research Support Center, School of Public Health and Family Medicine, Kamuzu University of Health Sciences, Blantyre, Malawi; 5 Kenya Medical Research Institute, Wellcome Trust Research Program, Nairobi, Kenya; 6 APIN Public Health Initiatives, Abuja, Nigeria; 7 Department of Paediatrics, College of Medicine, University of Lagos, Lagos, Nigeria; 8 Department of Paediatrics, Kamuzu University of Health Sciences (Formerly College of Medicine, University of Malawi), Blantyre, Malawi; 9 Kamuzu Central Hospital, Lilongwe, Malawi; 10 Department of Paediatrics and Child Health, Muhimbili University of Health and Allied Sciences, Dar Es Salaam, Tanzania; 11 Department of Curative and Medical Rehabilitation, Ministry of Health, Lilongwe, Malawi; 12 Department of Paediatrics and Child Health, University of Nairobi, Nairobi, Kenya; 13 Department of Paediatrics, College of Medicine, University of Ibadan, Ibadan, Nigeria; 14 Nuffield Department of Medicine, University of Oxford, Oxford, UK; 15 Department of Information Science, University of Otago, Dunedin, New Zealand; 16 Maternal and Newborn Health Group, Department of Infectious Disease Epidemiology and International Health, London School of Hygiene & Tropical Medicine, London, UK

**Keywords:** Newborn, Neonatal, Africa, Low- and middle-income countries, Quality of care, Health management information systems, Small and sick newborn care, Hospital records, Inpatient Care, Data for Action

## Abstract

**Background:**

Every Newborn Action Plan (ENAP) coverage target 4 necessitates national scale-up of Level-2 Small and Sick Newborn Care (SSNC) (with Continuous Positive Airway Pressure (CPAP)) in 80% of districts by 2025. Routine neonatal inpatient data is important for improving quality of care, targeting equity gaps, and enabling data-driven decision-making at individual, district, and national-levels. Existing neonatal inpatient datasets vary in purpose, size, definitions, and collection processes. We describe the co-design and operationalisation of a core inpatient dataset for use to track outcomes and improve quality of care for small and sick newborns in high-mortality settings.

**Methods:**

A three-step systematic framework was used to review, co-design, and operationalise this novel neonatal inpatient dataset in four countries (Malawi, Kenya, Tanzania, and Nigeria) implementing with the Newborn Essential Solutions and Technologies (NEST360) Alliance. Existing global and national datasets were identified, and variables were mapped according to categories. *A priori* considerations for variable inclusion were determined by clinicians and policymakers from the four African governments by facilitated group discussions. These included prioritising clinical care and newborn outcomes data, a parsimonious variable list, and electronic data entry. The tool was designed and refined by > 40 implementers and policymakers during a multi-stakeholder workshop and online interactions.

**Results:**

Identified national and international datasets (*n* = 6) contained a median of 89 (IQR:61–154) variables, with many relating to research-specific initiatives. Maternal antenatal/intrapartum history was the largest variable category (21, 23.3%). The Neonatal Inpatient Dataset (NID) includes 60 core variables organised in six categories: (1) birth details/maternal history; (2) admission details/identifiers; (3) clinical complications/observations; (4) interventions/investigations; (5) discharge outcomes; and (6) diagnosis/cause-of-death. Categories were informed through the mapping process. The NID has been implemented at 69 neonatal units in four African countries and links to a facility-level quality improvement (QI) dashboard used in real-time by facility staff.

**Conclusion:**

The NEST360 NID is a novel, parsimonious tool for use in routine information systems to inform inpatient SSNC quality. Available on the NEST360/United Nations Children's Fund (UNICEF) Implementation Toolkit for SSNC, this adaptable tool enables facility and country-level comparisons to accelerate progress toward ENAP targets. Additional linked modules could include neonatal at-risk follow-up, retinopathy of prematurity, and Level-3 intensive care.

**Supplementary Information:**

The online version contains supplementary material available at 10.1186/s12887-023-04341-2.

## Key findings

**Table:** 

**1. WHAT WAS KNOWN?**
• The Every Newborn Action Plan (ENAP) aims to reduce the 2.4 million newborn deaths per year and ensure newborns go on to thrive through the scale-up of Level-2 Small and Sick Newborn Care (SSNC) (with continuous positive airway pressure (CPAP)) in all countries. • Routine Health Information Systems (RHIS), such as DHIS (District Health Information Software), collect aggregate data. In some countries, these systems capture SSNC admissions and deaths. However, detailed indicators for quality of care (e.g., continuous positive airway pressure (CPAP) and treatment of serious neonatal infections) require individual-level data based on standardised datasets, which have been widely used in high-income settings for three decades. • Existing neonatal inpatient datasets are mainly developed for high-income contexts and vary in size, purpose, process, and context. Few publications detail the design process for an individual-level neonatal inpatient data tool for high-mortality settings. A standardised, parsimonious, individual-level neonatal inpatient dataset is required to enable quality improvement (QI) and learning networks in these settings.
**2. WHAT WAS DONE THAT IS NEW?**
• We applied a systematic approach in three steps to review, co-design, and operationalise a neonatal inpatient dataset to track neonatal outcomes and improve the quality of SSNC. • **Step 1—Review:** Neonatal ward routine data tools (i.e., patient forms/clinical case notes, registers, and aggregate summary forms) and datasets were identified and reviewed in countries (i.e., Malawi, Kenya, Tanzania, and Nigeria) implementing with NEST360 (Newborn Essential Solutions and Technologies). Further national (*n* = 1), international (*n* = 2), and study-specific (*n* = 3) individual-level datasets identified were also mapped by variable category. • **Step 2—Co-design:** The NEST360 Alliance Neonatal Inpatient Dataset (NID) *a priori* design considerations included: prioritising data on clinical care pathways and outcomes amenable to staff action on the newborn unit, a parsimonious essential variable list (~ 50 variables) per baby and supporting electronic data entry on-site. Through co-design, we selected 60 core variables in six categories: birth details and maternal history; neonatal admission details and identifiers; complications and observations; interventions/investigations; discharge outcomes, diagnoses or cause of death. • **Step 3—Operationalise:** The NEST360 NID has been operationalised in 69 units in 65 hospitals across four African countries (October 2019 to date) with differing case mixes, hospital levels, and health systems resources. The NID links via an electronic data flow pathway to produce a live NEST360 Quality Improvement Facility Dashboard for use by national and facility-level teams.
**3. WHAT WAS FOUND?**
• Identified individual-level inpatient datasets from high-mortality settings were primarily focused on research. Number of variables ranged from 55–254, and no datasets matched all *a priori* considerations. • Implementation lessons learned focused on the importance of co-designing with governments and embedded institutionalisation. For example, in Malawi, facility-based data collectors for data entry are government-employed, and the Ministry of Health hosts the local data server.
**4. WHAT NEXT?**
• The NEST360 NID tool is a global public good that can be adapted and used to support the 93 countries implementing ENAP targets to measure impact, coverage, and quality of Level-2 (with CPAP) SSNC. • The tool has been produced in paper and electronic formats to facilitate adaptation and use in other settings. The NEST360 NID can be interoperable and linked to DHIS. • Since the NEST360 NID was designed to be parsimonious to enhance data quality and data collection, there is a demand for additional modules in the future (e.g., follow-up after discharge, retinopathy of prematurity, infection outbreaks, and Level-3 intensive care).

## Background

Given that an estimated 2.3 million neonatal deaths occur globally annually, countries are focused on accelerating progress to meet Sustainable Development Goal 3.2, ending preventable deaths of newborns by 2030. The target of < 12 neonatal deaths per 1000 live births requires high coverage of Small and Sick Newborn Care (SSNC), including respiratory support for preterm babies. Hence, the *Every Newborn* Action Plan (ENAP), an UN-led multi-country partnership, agreed to targets for 2025, including the scale-up of Level-2 SSNC (with CPAP) [1–3] (Additional file 1). With > 80% of global births occurring in facilities [4], national and facility-level decision-makers require comparable newborn inpatient data relating to interventions, survival, and follow-up care to track national progress towards these coverage and quality of care targets [1].

Routine Healthcare Information Systems (RHIS) are a building block of a functioning health system [5]. Studies have shown that routine data use in high-mortality settings can improve coverage and quality of care [6]. However, in many of these settings, the lack of facility-level data for use impedes decision-making to accelerate preventable neonatal morbidity and mortality [5, 7, 8]. One example of RHIS is District Health Information Software, V.2 (DHIS2), now used widely in > 80 countries. Typically, health facility ward registers, often originating from paper tally sheets, serve as the primary source for collating aggregate data. This data is subsequently digitised at the facility level and elevated to national level databases [9]. However, to track quality of care for more complex clinical conditions, ward registers and tally systems cannot include all the required detail, especially for multi-faceted clinical denominators. This applies to many programmes, such as maternity care or non-communicable diseases and the more detailed individual-level neonatal care data [10–15].

In high-income settings, there is a long history of networks and research groups using standardised inpatient datasets – for example, Vermont Oxford Network [16, 17], a paid service operational in > 35 countries, including some LMICs such as South Africa. Many countries have national examples, such as the UK [18], USA [19] or Canada [20]. Datasets vary in the number of variables collected, purpose, definitions, collection process, and context. India’s Facility Based Newborn Care Database [21] and the Clinical Information Network (CIN) in Kenya [22] are also examples of established neonatal individual-level data collection programs in high-burden settings.

However, many of these existing neonatal inpatient tools were not parsimoniously designed and do not link to national data systems or focus on quality improvement in the Level-2 SSNC (with CPAP) ward. Furthermore, associated publications on these tools provide limited detail on their development, implementation, and institutionalisation.

NEST360 (Newborn Essential Solutions and Technologies) is a multi-partner alliance including national governments in four African countries (Malawi, Kenya, Tanzania, and Nigeria) with engineers, clinicians, and health systems implementation experts. The alliance aims to improve the quality of SSNC and foster learning by implementing a health systems package supporting ENAP targets and the WHO/UNICEF Quality of Care Network [23]. This intends to accelerate progress toward SDG 3.2, focused on neonatal survival.

The NEST360 Alliance set out to co-design and operationalise a core neonatal inpatient dataset tool to measure individual-level quality of care to inform quality improvement, measure impact and enable prompt action in neonatal units. This tool was intentionally designed to integrate with existing routine data systems (i.e., facility HMIS and DHIS) to enable sustainable use at scale in LMIC settings.

### Aim and objectives

This paper is part of a supplement reporting findings and learnings from NEST360, an alliance of partners, including four African governments (Kenya, Malawi, Nigeria, and Tanzania), working to reduce neonatal inpatient deaths by improving Level-2 newborn care in hospitals. In this paper, we aim to describe the systematic, evidence-based review, co-design, and operationalisation of a core neonatal inpatient dataset to improve the quality of care and track outcomes of small and sick newborns in high-mortality settings. This paper addresses three objectives:

- 1: Review of existing newborn ward data landscape across four countries implementing with NEST360 and describe other available study-specific and national or international neonatal inpatient datasets.
- 2: Co-design content of a novel, core dataset based on *a priori* considerations, with a focus on variables for quality improvement action, which are included in a small and sick newborn care facility-level dashboard.
- 3: Pilot, refine and operationalise a novel and core neonatal dataset in Malawi, Kenya, Tanzania and Nigeria, with intentional pathways to institutionalisation.

## Methods

NEST360 has produced a health systems package, including an innovative bundle of devices, data tools and a clinical education package. This has been implemented in 69 newborn units across 65 health facilities in Malawi, Kenya, Tanzania, and Nigeria (some hospitals have geographically separated inborn and outborn units).

**Fig. 1:**
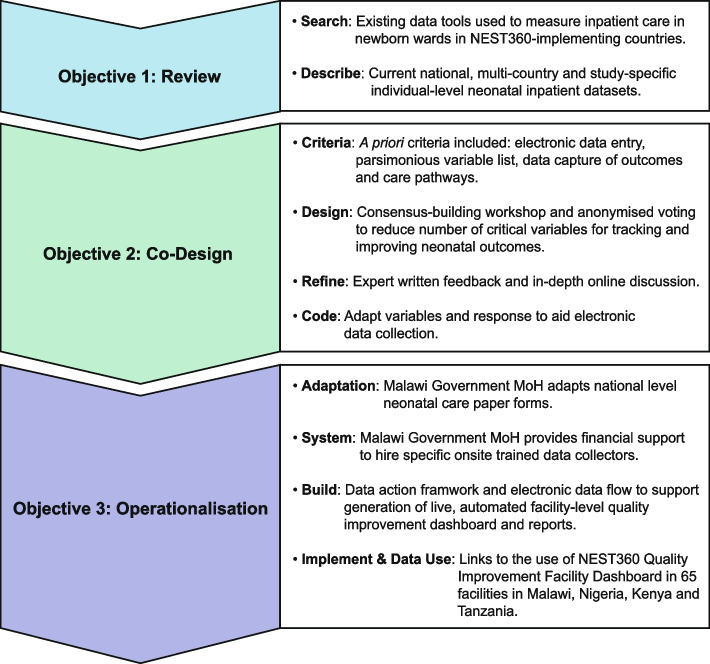
Three-step systematic, evidence-based, objective framework used to review, co-design and operationalise the NEST360 Neonatal Inpatient Dataset. Abbreviations: MoH, Ministry of Health; NEST360, Newborn Essential Solutions and Technologies

As part of this package, we applied a systematic evidence-based developmental approach to the review, co-design, and operationalisation of the NEST360 Neonatal Inpatient Dataset (NID) tool [24]. This was conducted in a three-step objective framework (Fig. 1). This process took place from June 2019 to January 2022.

### Methods by objectives

#### Objective 1: Review of existing newborn ward data landscape across four countries implementing with NEST360 and describe other available study-specific and national or international neonatal inpatient datasets

Design of the NID data tool began with stakeholder consultation. Relevant data forms, clinical case notes or registers used in the newborn ward in countries implementing with NEST360 were identified and described.

Identified international, national, and study-specific individual-level datasets used in other LMICs were mapped with each variable (i.e., initial data element within a path of branching logic) as a row and each dataset as a column in Microsoft Excel (Microsoft Corporation, USA). Datasets were described by the number and proportion of total variables by category. The Standards for Improving the Quality of Care for Small and Sick Newborns in Health Facilities were unavailable at this stage of development but were cross-checked after publication in 2020 [25].

#### Objective 2: Co-design content of a novel, core dataset based on *a priori* considerations, with a focus on variables for quality improvement action, which are included in a small and sick newborn care facility-level dashboard

Building on the review of pre-existing individual-level data tools described in Objective 1, a face-to-face workshop was conducted in Nairobi, Kenya (June 2019). Multi-stakeholder participants contributed, including study implementers, clinicians, nurses, and Ministry of Health officials (i.e., information system departments) from countries implementing with NEST360 (i.e., Malawi, Kenya, Tanzania, and Nigeria) (Additional file 2). Facilitated group discussions in English were conducted to explore differences and similarities for settings where the NID would be implemented. Variable selection for inclusion into the NID was informed by the data needed to support quality improvement in the newborn ward. A draft design of a quality improvement facility dashboard (i.e., a graphical user interface used to view key performance indicators) focusing on the newborn ward was formed during initial discussions to aid selection of specific variables to action its use. Anonymised electronic voting questionnaires (Mentimeter™, Mentimeter AB, Stockholm, Sweden) were used to reduce the initial list of variables in each category. More than 75% was considered a majority vote. Meeting moderators presented the findings and comments for those variables that garnered ≤ 75% agreement from the questionnaire responses. The voting process was repeated after facilitated discussion to reach a consensus when necessary. The resulting tool was shared with participants to allow further corrections and reflections. In addition, feedback was obtained from researchers at the London School of Hygiene & Tropical Medicine (LSHTM, UK), Rice University (USA) and the University of Oxford (UK). These teams contained expertise in clinical care (including a focus on newborn healthcare), routine data systems, programming, epidemiology, data management and statistics. Web-based conference calls followed, and written email feedback was provided in line with dataset variables.

The data tool was refined using the development of branching logic and database validations to simplify data entry and improve data quality. The NID data tool was compared with current WHO standards and guidelines for small and sick neonatal care and revised [25]. Paper case report forms were drafted, reviewed, and refined to align with specific variables within national forms. These forms were developed to support use in areas where internet connectivity is inconsistent (Additional file 3).

#### Objective 3: Refine and operationalise a novel and core neonatal dataset in Malawi, Kenya, Tanzania and Nigeria, with intentional pathways to institutionalisation

Data governance is key to NID operationalisation, with data servers hosted locally and owned by the Ministry of Health in most countries. The NID and its operating procedures support all appropriate General Data Protection Regulations (GDPR) and WHO data principles [26]. Data obfuscation of all identifiable information (e.g., names, phone numbers, and address notes/directions) occurs within facility-level or country-level servers before data are shared with the NEST360 Alliance, as described in signed data sharing agreements.

NEST360 and Ministry of Health data collectors were trained between March 2020 and April 2021 (not applicable to Kenya, as NEST360 NID links to Clinical Information Network (CIN) data collection systems). This process used a Training of Trainers (ToT) cascade model, with the NEST360 country data manager being the lead trainer, promoting country leadership and ownership. ToT sessions were mostly given during the COVID-19 pandemic using online videoconferencing software. Training focused on attaining skills, with sessions relating to NEST360 and NID objectives, data collection procedures, data quality assessment and competency-based use of the NID data tool and associated devices. Data entry practice with standardised cases and online pre- and post-training evaluations confirmed skill and knowledge attainment. Neonatologists and neonatal nurses also attended to provide context, acquire knowledge, and supply line-by-line explanations of the data elements in the tool.

The NEST360 NID dataset was developed within a REDCap database (Research Electronic Data Capture, Vanderbilt University, TN, USA) hosted in each NEST360 country server using a set of standard operating procedures and data flow scheme (Additional file 4). All data management, reporting, and output scripts were developed in Stata version 17™ (StataCorp LLC, Texas, USA) and R (R Foundation for Statistical Computing, Vienna, Austria) software™.

## Results

Our process description and learnings are summarised according to the three-objective framework (Fig. 1) as follows:

### Objective 1: Review of existing newborn ward data landscape across four countries implementing with NEST360 and describe other available study-specific and national or international neonatal inpatient datasets

A review of the data landscape used across Level-2 (with CPAP) newborn units in the four countries implementing with NEST360 led to the identification of 21 tools (Table 1). This included mapping eight patient forms, eight registers and five aggregate data sheets. Standardised Ministry of Health (MoH) individual-level patient forms are used within some facilities in Malawi and Kenya. However, all countries lacked a nationwide neonatal inpatient dataset. Documented national registers focus on Kangaroo Mother Care (KMC), CPAP, postnatal care, and inpatient admissions. Data tools collecting newborn data outside of the newborn ward were not included in this review, as they were not deemed source documents critical to actioning care quality improvement in the newborn ward. Data tools used specifically in KMC wards and corners were also not included.

**Table 1:** Review and mapping of existing national routine data landscape in the newborn ward across countries implementing with NEST360

**Country**	**Category**	**Document Name**	**Purpose**	**No. Variables**	**No. Facilities**	**Years**	**Level**	**Overview**
Kenya^a^	Register	Inpatient Neonatal Register	Standardised National MOH	41	221	2020—Present	Individual	Captures information on demographics, diagnosis, key interventions, and outcome for all newborns admitted to newborn units.
	Register	KMC Register	Non-Standardised Hospital-Specific	23	^b^	^b^	Individual	Completed in KMC ward for the admitted babies.
	Register	CPAP Register	Non-Standardised Hospital-Specific	13	^b^	^b^	Individual	Completed within the newborn unit for babies admitted and receiving CPAP management.
	Patient Form (Paper)	Neonatal Admission Record	Standardised National MOH	80	221	2006—Present	Individual	Used to capture triage data on each inpatient. Divided into sections: 1) maternal history, 2) neonatal biodata and clinical history, 3) neonatal examination findings and admission vital signs, 4) basic laboratory tests ordered and 5) primary and secondary diagnosis on admission [27].
	Patient Form (Paper)	Comprehensive Newborn Monitoring Form	Research (CIN)	43	24	2019—Present	Individual	Used to capture inpatient data on interventions and monitoring delivered to admitted newborns [28].
	Patient Form (Paper)	Newborn Unit Discharge/Exit Form	Research (CIN)	20	27	2018—Present	Individual	Summary information that includes demographics, discharge diagnosis and outcome.
	Patient Form (Paper)	Internal Newborn Unit Transfer Form	Research (CIN)	56	24	2019—Present	Individual	Complete for all newborns in maternity requiring admission to the newborn unit
	Aggregate Data	Inpatient Neonatal Summary	Standardised National MOH	54	221	2019—Present	Facility	Captures aggregate neonatal data relating to admissions, birthweight, admission weight, deaths, cause of admission, gestation at birth, interventions, and deaths due to specific conditions.
Malawi	Register	National KMC Register	Standardised National MOH	5	195	2015 – Present	Individual	A national-level register capturing five indicators: 1) KMC initiation rate, 2) KMC referral completion, 3) survival to discharge, 4) death before discharge, 5) left against medical advice [8].
	Register	National Sick Neonate Register	Standardised National MOH	21	195	2017 – Present	Individual	Documentation of each admitted baby and monthly admission summaries.
	Patient Form (Paper)	Neonatal Admission Form	Standardised National MOH	50	195	2018 – Present^c^	Individual	Two pages (i.e., labour ward and nursery ward) completed at the time of admission.
	Patient Form (Paper)	Critical Care Pathway Form	Standardised National MOH	37	195	2018 – Present^c^	Individual	Monitoring sheet for each admitted newborn throughout the admission duration in the nursery ward (inc. vitals, daily weight, interventions, and drug administration).
	Patient Form (Paper)	Acute Respiratory Illnesses Form	Research (CPAP Study Team)	50	36	2013 – 2019	Individual	Form used to collect information relating to CPAP or oxygen therapy use [29].
	Patient Form (Paper)	CPAP Monitoring Sheet	Standardised National MOH	26	36	2013 – Present	Individual	Monitoring sheet for newborns on CPAP including machine settings, vitals, and signs of respiratory distress [29].
	Aggregate Data	Sick Neonate – Facility Monthly Report	Standardised National MOH	29	37	2021 – Present	National	Output data in DHIS2 from the Facility Monthly Report Form.
	Aggregate Data	Maternity Monthly Report	Standardised National MOH	47	195	2016 – Present	National	Data relating to five categories: 1) delivery process, 2) delivery location, 3) maternal status, 4) newborn complications, 5) newborn outcomes.
Tanzania	Register	Postnatal Register	Standardised National MOH	33	6602	2018—Present	Individual	Filled during postnatal care visits for mother and baby within 42 days after delivery.
	Register	National KMC Register	Standardised National MOH	24	2534	2016—Present	Individual	Filled at KMC ward for the newborns admitted at KMC ward and the services received during hospital stay.
	Aggregate Data	Tally Postnatal	Standardised National MOH	3	6602	2018—Present	Facility	Used together with the postnatal register to ensure all information entered into the register is entered in the appropriate section.
	Aggregate Data	Postnatal Summary Report Form	Standardised National MOH	4	6602	2018 – Present	Facility	Filled during the first week of the following month and copy sent to district to be entered into DHIS2.
Nigeria	Register	Neonatal Admission Register	Standardised FMOH / SMOH	35	5865	2015 – Present	Individual	Used in each newborn ward to capture data relating to the newborn admitted to the unit, including variables relating to demographics, delivery information, diagnosis, interventions, and outcome.

Data tools used outside the newborn ward to collect neonatal data were not included in this review

*CIN* Clinical Information Network, *MOH* Ministry of Health, *No.* Number, *DHIS2* District Health Information Software 2, *CPAP* Continuous Positive Airway Pressure, *KMC* Kangaroo Mother Care, *FMOH* Federal Ministry of Health, *SMOH* State Ministry of Health

^a^A more detailed description of other data tools used outside of the newborn ward to collect newborn data can be observed in Hagel et al*. *[30]

^b^These are non-standardised registers that are hospital-specific, as result number of facilities and start date could not be provided

^c^With respect to the current version of the form

A total of six individual-linked datasets used in LMIC settings were identified through expert and stakeholder consultation (Table 2). Tools were analysed regarding the number of variables, purpose, and variable categories dedicated to a topic. Across all tools, these included maternal history, birth details, admission details and identifiers; newborn complications and observations; newborn interventions, discharge outcomes, diagnosis or cause of death, and post-discharge follow-up. The number of variables across datasets had a median of 89 (interquartile range: 61 to 154). All datasets varied by purpose, including research, quality improvement, monitoring specific interventions (e.g., CPAP, KMC, nutrition), data quality, and tracking outcomes.

The Vermont Oxford Network (VON) Global Neonatal Database (piloted by the Ethiopian Neonatal Network) is incorporated within a series of prepaid service packages with technical support, online data management and educational materials [31]. As an essential component of the rapid scale-up of Special Newborn Care Units (SNCU) in India (i.e., expanding Level-2 care to district facilities), India’s Facility Based Newborn Care Database is specific for neonatal data and is being implemented across 934 units, which admit over 1.3 million newborns annually [21]. Trained data collectors use a standardised form for digital entry that contains parameters relating to demographic, anthropometric and admission data, treatments, and outcomes. The Clinical Information Network (CIN) is a research partnership between the KEMRI-Wellcome Trust Research Programme, the Kenyan Paediatric Association, and the Ministry of Health. Their dataset includes 120 variables to examine outcomes and improve data quality and is now used to identify service delivery problems [22].

Maternal history variables were commonly found within the Indian SNCU dataset (36% of total variables) and the Neonatal Nutrition Network (NeoNuNet) (33%). The NeoNuNet and VON datasets held few variables relating to admission details (NeoNuNet: 12%, VON: 9%) and newborn complications and observations (NeoNuNet: 1%, VON: 2%). WHO iKMC research study dataset was the largest (*n* = 254) designed to focus heavily on a specific intervention, similar to the CPAP Data Summary dataset.

### Objective 2: Co-design content of a novel, core dataset based on *a priori* considerations, with a focus on variables for quality improvement action, which are included in a small and sick newborn care facility-level dashboard

Nairobi Workshop attendees (including Ministry of Health colleagues) agreed on *a priori* considerations for a proposed NID, including:

- Variables focused on Level-2 SSNC (with CPAP) clinical care pathways and outcomes that are measurable, high-impact and actionable in the newborn ward for quality improvement, and input into a facility-level dashboard.
- Parsimonious variable list (aiming for ~ 50 variables per baby)
- Electronic data entry (at the time of discharge)

Attendees reviewed all 300 collated variables from the initial mapping of the datasets (Additional file 5). This list was reduced to approximately 100 variables through anonymised voting by comparing against *a priori* considerations. A list of 55 variables was agreed upon using further voting and consensus-building processes. Examples of eliminated variables included maternal history variables (i.e., gravidity and parity), which attendees noted were important, but could not be acted upon in the newborn ward. Maternal variables with a direct impact on the management of sick newborns, including premature rupture of membranes, maternal fever, age, status, cause of death, mode of delivery, antenatal corticosteroids, HIV status and chronic conditions (i.e., diabetes and hypertension), were included in the NID tool. Variables relating to study-specific initiatives (e.g., in-depth descriptions of IV fluids, nutritional supplements, and multiple recurrent weight observations for each neonate) were also removed due to collection difficulty and the lack of relevance to routine neonatal inpatient care. Finally, some variables (e.g., best obstetric, or neonatal gestational age assessment, neonatal blood culture results) were considered challenging to collect accurately. However, due to their importance to clinical care, they were included in the NID, with the recognition that data quality and utilisation could be improved over time by measuring them.

In the following year, the NEST360 Neonatal Inpatient Dataset Learning Group revised the dataset variables through online discussions and written email feedback. Written feedback included integrating a standardised record ID, enhanced KMC variables, a new readmission variable, identifiers for multiple births, and enhanced geospatial data. This expanded the dataset to encompass a total of 60 variables (Table 2).

**Table 2:** Description of identified neonatal individual-level datasets by variable categories

**Type**	**Individual-Level Datasets**						
**Tool**	**Neonatal Nutrition Network**	**Clinical Information Network**^c^	**CPAP Data Summary**^d^	**WHO iKMC Study**	**SNCU Facility Based Newborn Care Database**	**Vermont Oxford Global Database**	**NEST360 Neonatal Inpatient Dataset**
**Reference**	[32]	[27]	[29]	[33, 34]	[21]	[31]	-
**Country**	Nigeria	Kenya	Malawi	Multi-Country	India	Multi-Country	Multi-Country
**No. Variables**	69	120	55	254	113	64	60
**Purpose**	Research (Newborn Nutrition)	Research (Monitoring Data Quality)	Research (Oxygen Therapy and CPAP Use and Outcome)	Research (KMC Use and Outcome)	Programme (Monitoring Outcomes)	Programme (Quality Improvement)	Alliance (Quality Improvement and Tracking Outcomes)
**Category**	**Number of Variables (Proportion of Variables, %)**						
Maternal History^a^	23 (33%)	19 (16%)	4 (7%)	67 (26%)	41 (36%)	14 (22%)	6 (10%)
Birth Details	7 (10%)	9 (8%)	8 (15%)	27 (11%)	14 (12%)	13 (20%)	8 (13%)
Admission Details and Identifiers	8 (12%)	25 (21%)	14 (26%)	28 (11%)	17 (15%)	6 (9%)	14 (23%)
Newborn Complications and Observations	1 (1%)	25 (21%)	13 (24%)	56 (22%)	32 (28%)	1 (2%)	11 (18%)
Newborn Interventions^b^	16 (23%)	34 (28%)	13 (24%)	49 (19%)	2^e^ (2%)	14 (22%)	13 (22%)
Discharge Outcomes, Diagnosis, Cause of Death	14 (20%)	7 (6%)	3 (6%)	21 (8%)	7 (6%)	16 (25%)	7 (12%)
Post Discharge Follow-up	0 (0%)	1 (1%)	0 (0%)	6 (2%)	0 (0%)	0 (0%)	1 (2%)

*Abbreviations*: *NEST360* Newborn Essential Solutions and Technologies, *ARI* Acute Respiratory Illnesses, *KMC* Kangaroo Mother Care, *CPAP* Continuous Positive Airway Pressure, *WHO* World Health Organisation, *SNCU* Special Newborn Care Unit, *IV* Intravenous, *CIN* Clinical Information Network

^a^Includes variables relating to maternal history (i.e., chronic, infection, obstetric), family and social economic background

^b^Module contains variables relating to microbiology, KMC, Oxygen therapy, CPAP, mechanical ventilation, antibiotics, apnoea treatment, photography, IV fluids, and transfusions

^c^Using CIN REDCap data dictionary (2019)

^d^Using Acute Respiratory Illness Form

^e^Both variables are open-ended note format data types

In March 2020, the COVID-19 pandemic led to the addition of a module to capture variables relating to maternal and newborn COVID-19 symptoms and test results. The COVID-19 module was designed to aid the descriptive analysis of neonatal outcomes by maternal COVID-19 status. Content was guided by ISARIC-WHO Case Report Forms, early publications, and expert review [35–38]. In addition, this module was aligned to ongoing clinical trials to support analogous COVID-19 case definitions (i.e., OMWaNA Trial) [39]. At this time, the NID was also aligned with an ongoing registry being used in three Nigerian tertiary facilities [40]. The dataset’s modular design and simplistic coding style facilitate modifying or removing these variables at any stage, ensuring adaptability to evolving needs.

Code development of the dataset involved selecting a software programme and designing a branching structure. Our team had previous experience using REDCap, KoBo Toolbox (KoBo Inc., Cambridge, MA, USA) and Open Data Kit (ODK, San Diego, CA, USA). Though not open-source, REDCap was chosen due to enhanced data security (e.g., custom user rights and password protection), ease of use (e.g., limited programming experience needed), flexibility (e.g., offline and online use), linkage (e.g., data synchronisation using application programming interface keys) and in-built validation fields (e.g., weights and dates). The NEST360 Alliance agreed to start development with this software but move to an open-source platform in the future. A standardised REDCap data dictionary was formatted with consistent variable naming, module structure, variable formats, and reduced free text to aid user-friendly data collection, support data quality assessments, and enable future adaptation (Additional file 6).

**Fig. 2:**
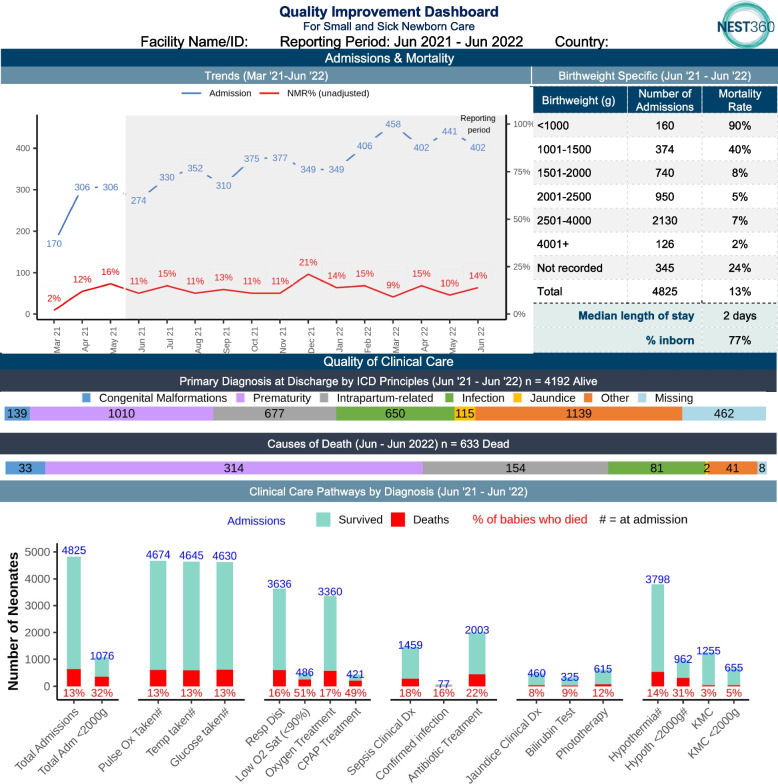
NEST360 Quality Improvement Facility Dashboard (Phase 1 – Paper Format). The bottom third section of the dashboard is not generated from data collected by the NEST360 Neonatal Inpatient Dataset, and as a result, it is not highlighted here. This is the current dashboard design at the time of publication, regular updates are planned. Abbreviations: ID, Identification; NEST360, Newborn Essential Solutions and Technologies; Adm, Admission; Ox, Oximetry; Temp, Temperature; Resp Dist, Respiratory Distress; O2 Sat, Oxygen Saturation; CPAP, Continuous Positive Airway Pressure; QI, Quality Improvement; Dx, Diagnosis; Hypoth, Hypothermia; KMC, Kangaroo Mother Care; NMR, Neonatal Mortality Rate; ICD, International Classification of Diseases

Regarding real-time data use by facility staff, the NEST360 Alliance has learnt from the design, ongoing refinement, and implementation of the NEST360 Quality Improvement Facility Dashboard (Fig. 2). Early dashboard designs informed the focus of the NID variables, prioritising data to inform quality improvement. The dashboard was developed using an open-source platform in RShiny [41] and automated using the integrated data flow process to ensure data are displayed in real-time, with interactive data visualisations (Additional file 4). The dashboard shows three organised Donabedian model components: admissions and mortality (i.e., outcomes), quality of clinical care (i.e., process), and health system inputs (i.e., enabling environment).

### Objective 3: Refine and operationalise the novel and core neonatal dataset in Malawi, Kenya, Tanzania and Nigeria, with intentional pathways to institutionalisation

While operationalising the NID on a pathway to institutionalisation at facility and national-levels, insights have been gained across multiple countries. These implementation insights focus on two main themes: human resources and data collection processes.

#### Human resources

As learnt in other neonatal data collection processes [21], a dedicated data collector is crucial to aid timely data collection, improve data quality, and reduce workload for the clinical staff, especially nurses. The Malawian Government Ministry of Health (MoH) has implemented this practice along with redeveloping national-level neonatal inpatient forms (e.g., Neonatal Admission Form (NAP) and Critical Care Pathway (CCP) Form) to align with NEST360 NID variables. No standard nationwide neonatal inpatient forms or clinical case notes were available in Tanzania or Nigeria during the initial operationalisation phase of the NID (Table 3).

**Table 3:** NEST360 Neonatal Inpatient Dataset integration into country and facility existing data flow systems

**Details**	**Malawi**	**Nigeria**^f^	**Tanzania**	**Kenya**
Number of Facilities Implementing with NEST360	36	9	7	13
Patient Form(s)	(1) Neonatal Admission Form (2) Critical Care Pathway Form	Facility-Specific	Facility-Specific	(1) Neonatal Admission Record (2) Comprehensive Newborn Monitoring Form (3) Internal Transfer Form (4) Newborn Unit Discharge/Exit Form
Patient Forms Adapted to Accommodate NEST360 NID Collection	Yes^c^	No	No	Yes^g^
NID Form	Hybrid (Electronic and Paper)	Paper	Electronic	Electronic
Data System^a^	REDCap	REDCap	REDCap	REDCap
Current Data Management Location^b^	Kamuzu University of Health Sciences^d^	APIN Public Health Initiatives	Ifakara Health Institute	KEMRI-Wellcome Trust (CIN Project Database)
Future Data Management Location	MOH^e^	MOH^e^	MOH^e^	KEMRI-Wellcome Trust

*Abbreviations*: *NEST360* Newborn Essential Solutions and Technologies, *DHIS2* District Health Information Software 2, *NID* Neonatal Inpatient Dataset, *REDCap* Research Electronic Data Capture, *CIN* Clinical Information Network, *KEMRI* Kenya Medical Research Institute, *MOH* Ministry of Health

^a^REDCap is a secure, web-based software platform designed to support data capture for research studies [42]

^b^With data pooling at LSHTM

^c^Adapted by Malawian Ministry of Health

^d^At the point of publication, with actions underway to locate at Malawi MOH

^e^With linkage to DHIS2 [9]

^f^National neonatal data harmonisation is ongoing

^g^Adapted data dictionary by CIN [27]

#### Data collection processes

NID data collection is a systematic process contingent upon the available health system resources. The process begins upon the discharge of a newborn, where a facility-based data collector is responsible for collecting individual-level data from primary source documentation such as patient forms or clinical case notes. This information is then utilised to summarise the admission and the care the newborn receives. In instances where data is missing, the collector engages in dialogue with clinical and laboratory teams to rectify. In cases where data cannot be located or read, the collector will indicate “not recorded” or “not readable” to indicate areas where clinical documentation could be improved.

The mode of data entry can vary across settings; some opting to initially enter data onto paper and subsequently transcribe this data into the REDCap project database, whereas others prefer to input data directly into the database. This decision is often contingent upon the availability of internet connectivity and space within the unit. Utilising a desktop-based entry system with a dedicated data collector within the unit has improved data timeliness compared to utilising paper entry on-site (with off-site database entry), as demonstrated initially in Malawi. The time delay between data collection and entry was significantly reduced, from a median of 49 days (IQR:6–120) to 6 days (2–23) (Additional file 7). A desktop-based system also provides advantages such as greater processing power, internet access, and a larger screen interface. Moreover, users have reported that a physical keyboard enhances ease of use compared to alternative input methods. Despite this, hard-copy data collection is preferred in some facilities to aid local data audit cycles. Data collectors regularly receive periodic data query lists generated by automated R scripts that analyse the dataset for errors related to completeness and accuracy. Additionally, these scripts assess the timeliness of data entry for ongoing review (Additional file 7).

## Discussion

A standardised national-level neonatal dataset is important for high-quality newborn clinical care, but such datasets are lacking for use in contexts where most neonatal deaths occur [43]. Of the neonatal inpatient datasets we identified from high mortality settings, the size ranged from 55–254 variables and did not match predefined NEST360 Alliance *a priori* design considerations. Such a tool must be parsimonious to increase wider and more sustainable use. Therefore, we applied a systematic approach with four African governments and country teams to review, co-design, and operationalise a dataset to track clinical care processes and outcomes for high-quality SSNC. The NEST360 NID currently comprises 60 variables and has been operationalised in 69 units in 65 hospitals across Malawi, Kenya, Tanzania, and Nigeria with differing bed capacities, case mix, and human resources. To date (late 2023), data for over 450,000 newborns have been captured. This NID tool can potentially address the inverse data law, where the largest burden contexts have the least data [44].

Four of the six individual-level datasets identified from LMIC settings were primarily designed for research, while two focused on quality monitoring and improvement (e.g., Vermont Oxford’s Global Neonatal Database and Clinical Information Network (CIN)) [27, 31]. The remaining dataset was dedicated to monitoring outcomes, as seen in India’s Special Newborn Care Unit Database [21, 31]. Upon mapping the variables within these existing databases, significant variability was observed due to these differing purposes.

The NID tool comprises six categories: birth details and maternal history; neonatal admission details and identifiers; complications and observations; interventions/ investigations (including microbiology); discharge outcomes, diagnosis or causes of death. Compared to others, one notable feature of the NID tool is the selective inclusion of maternal variables, such as a history of diabetes and hypertension. Our co-design process emphasised the importance of minimising the data collection burden whilst enabling quality improvement in the newborn ward and individual-level tracking of critical Level-2 (with CPAP) interventions and outcomes.

High-quality data entry is crucial for maintaining accurate and reliable information in healthcare settings. Electronic systems with in-built validation fields and branching logic can greatly enhance the accuracy and efficiency of data entry. However, optimal data quality can be challenging to achieve without a dedicated government-employed data entry collector in neonatal wards equipped with a designated desktop computer for real-time data input. An example of this strategy’s effectiveness is the scale-up of India’s national SSNC program (as of 2023, comprising 934 units admitting approximately 1.3 million patients annually). Implementing dedicated data entry collectors within these units has significantly improved data quality and timeliness [45–48]. This task-shifting approach supports WHO recommendations to strengthen and expand the health workforce and has been observed to reduce nurse workload [49]. Within the NEST360 Alliance, the Malawian Ministry of Health has strategically invested in human resources by financing the deployment of government data collectors across all 38 implementing units. These data collectors are employed and paid for by the Ministry of Health and utilise internet access provided by the Ministry, ensuring their long-term sustainability (Additional file 8). The initiative was established based on national learning and has supported the nationwide scale-up of neonatal care [29, 50]. Similarly, other governments implementing with the NEST360 Alliance have embarked on a similar trajectory, albeit at a later stage. In June 2023, Tanzanian facilities plan to transition the designated data collectors in each newborn unit to their payroll.

Data quality of the NID is further enhanced by incorporating regular embedded data quality checks, which generate data quality heatmaps in automated reports. These reports facilitate facility and national-level data meetings, promoting informed decision-making within newborn units. In addition, actions are managed locally, with on-site data collectors promptly addressing data queries.

Using high-quality data is an important component of supporting quality improvement [51]. However, the “Data rich, information poor” (DRIP) syndrome [52] may pose a risk if numerous variables are collected without analysis and use. Within NEST360, a balance has been achieved by linking electronic NID data collection to a real-time quality improvement facility dashboard generation via a secure data flow pathway (Additional file 4). The dashboard facilitates nationally-owned quality improvement processes at facility-level, supported by associated reports [53]. These interconnected outputs have proven useful in identifying and monitoring issues such as low CPAP and blood culture usage, high rates of hypothermia on admission, and gaps in newborn data (e.g., birthweight-specific mortality).

**Fig. 3:**
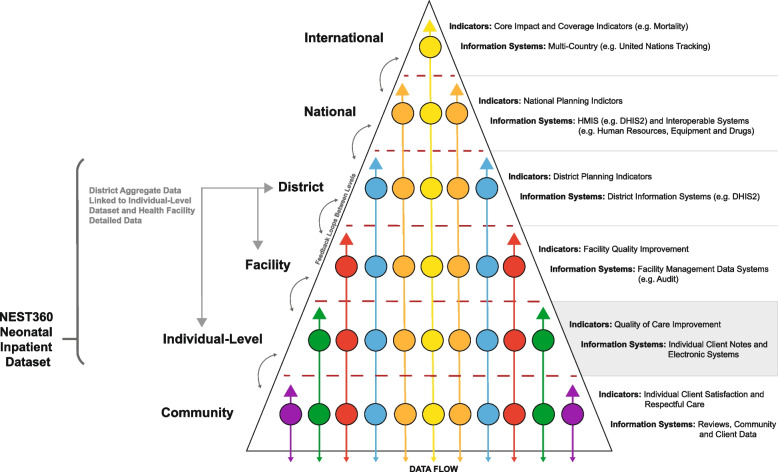
Neonatal data collection and use by health system level, capturing specific indicators using specific information systems. Adapted from [48, 54, 57]. Colours are illustrative of indicators but are generic and do not refer to specific indicators. Abbreviations: HMIS, Health Management Information Systems; DHIS2, District Health Information Software 2

Integration with national Routine Health Information Systems (RHIS) is crucial for large-scale, sustainable use, a factor consistently considered throughout the NID co-design process. Facility-level count data gathered from labour or newborn ward registers are compiled into monthly tally sheets. These aggregate data are then passed to district and national-level electronic platforms such as DHIS, which is utilised in over 80 LMICs [9]. For example, admissions and deaths count data on a newborn ward can be included as indicators within the RHIS/DHIS system, providing valuable facility-level admissions and outcome data [48, 54, 55]. The individual-level NEST360 NID can now connect to the system, enabling more comprehensive analyses, including tracking high-impact intervention coverage and quality using more complex denominators based on clinical characteristics (e.g., CPAP or KMC) and informing quality improvement at all levels [54, 56]. This interoperable data system design (Fig. 3) allows for reliable and timely data on detailed care without overwhelming the RHIS/DHIS2 system with multiple indicators that may not be feasible in tallied aggregate data.

Within the NEST360 Alliance, the development of the NID has also supported the improvement of existing routine national-level neonatal care forms. For example, the Malawi government modified their standard paper Neonatal Admission Form (NAP) to align with key variables collected using the NID. Similarly, in Tanzania, newly designed routine forms are being adapted to incorporate NID variables. In addition, the NID data collected in Malawi is also transitioning to being hosted on the government DHIS2 server platform. This interoperable nature of NID with other systems and existing forms has resulted in reduced workloads and intentionally institutionalised the tool within national government-owned information systems, which is critical for its long-term sustainability and fostering local capacity development within the public health sector [58, 59].

A significant strength of the NEST360 NID development lies in its collaborative co-design approach and the adoption of a transparent, evidence-based three-step systematic process. This consensus-driven methodology has proven effective in various applications [8, 60] and is recommended by a reporting guideline for the development of tools to assess performance management [61]. *A priori* NID design considerations set with government colleagues and implementers included: electronic data entry (emphasising local data ownership as a fundamental principle), a parsimonious design (ideally ~ 50 variables per admitted newborn), and prioritising data collection on clinical care pathways and outcomes that directly contribute to quality improvement action on the newborn unit. In addition, the co-designed tool undergoes continuous refinement, informed by implementation learning, review, and feedback from facility and country-level users. To date, it has been implemented in 65 facilities across four African countries, reflecting its practical utility and substantial impact in healthcare facilities.

There are limitations to this process. Our review may have missed other datasets if unpublished or not openly accessible. Datasets confined to single facilities or those primarily focused on maternal care aspects were not included [62–65]. Comparisons between datasets were complex due to variable interpretation, sub-branch responses, and variable categorisation, although our review consistently counted only initial branches as distinct variables. The NID data system is coded in REDCap, necessitating a license; however, a transition to open-source software is planned. Automated data quality checks and linked quality improvement facility dashboards are all coded in open-source systems, aiding accessibility and adaptability. The Malawian Ministry of Health has made a significant strategic investment at national-level, facilitating the employment of on-site data collectors in 38 facilities. Concurrently, healthcare facilities in Nigeria, Tanzania, and Kenya have received some financial assistance through research and programme initiatives in collaboration with local governments, supporting the employment of data collectors and ensuring data accessibility and use for government departments. To guarantee long-term sustainability, governments must continue to integrate the NID data collection process into their healthcare systems, ensuring the provision of these essential resources in the future.

The NID is purposefully designed to enhance data-driven action for Level-2 facilities (with CPAP) in LMIC contexts; hence, there is an intentional lack of variables for Level-3 SSNC (e.g., advanced feeding support such as screening for retinopathy of prematurity, TPN, and paediatric surgery). Future adaptations could involve the reduction or expansion of the tool, for example, for use in humanitarian settings [66] or Level-3 neonatal intensive care units, respectively. One important additional module already suggested in the co-design process was the follow-up of at-risk newborns after discharge, especially after preterm birth [67], severe bacterial infections [68], jaundice [69], and neonatal encephalopathy [70]. The innovative Indian SNCU dataset incorporates this feature, employing an SMS link to community workers for follow-up. Ensuring follow-up and data linkage is critical for neonates at risk of prematurity retinopathy. Consequently, an add-on NID module targeting this component of SSNC will be made available within the next update. Furthermore, a critical next step involves conducting a comprehensive costing analysis of NID implementation, detailing financial expenses related to personnel, hardware, software, and internet connectivity. This analysis will enable further incorporation of the NID process within other routine district hospital systems, promoting its long-term integration and use.

## Conclusions

In conclusion, the NEST360 NID has the potential to be used widely by facilities and countries to improve care and benchmark with others. However, ongoing institutionalisation within government systems is critical for wider use and improving the quality of data and care, which is the focus of the NEST360 Alliance. Therefore, we will continue to use and refine this tool and the linked quality improvement facility dashboards and processes. We welcome adaptation, feedback, and partnerships with others as we share the common aim of improving the quality of clinical neonatal care worldwide and accelerating progress to reduce the annual toll of 2.3 million neonatal deaths.

## Supplementary Information

**Additional file 1. **WHO Levels of Newborn Care with Interventions - NEST360 Informing the ‘How To’ for Implementation.

**Additional file 2.** Summary list of Nairobi 2019 workshop participants by characteristic.

**Additional file 3. **Neonatal Inpatient Dataset paper tool.

**Additional file 4.** Neonatal Inpatient Dataset data flow.

**Additional file 5. **Summary list of 300 potential variables for inclusion into the NEST360 Neonatal Inpatient Dataset, discussed at Nairobi 2019 workshop.

**Additional file 6.** Neonatal Inpatient Dataset REDCap data dictionary.

**Additional file 7.** Evaluating facility-level data entry timeliness in Malawi, Kenya, Tanzania, and Nigeria.

**Additional file 8.** NEST360 Neonatal Inpatient Dataset data collection infrastructure by facility.

**Additional file 9.** Local ethical approval for the complex evaluation of the implementation of a small and sick newborn care package with NEST360.

## Data Availability

Data sharing and transfer agreements were jointly developed and signed by all collaborating partners in the NEST360 Alliance. The NID tool, data dictionary and associated materials are available from NEST360/UNICEF Implementation Toolkit for Small and Sick Newborn Care and NEST360 website [24, 71].

## Abbreviations

CIN: Clinical Information Network
CPAP: Continuous Positive Airway Pressure
DHIS: District Health Information Software
ENAP: Every Newborn Action Plan
HMIS: Health Management Information Systems
ISARIC: International Severe Acute Respiratory and Emerging Infection Consortium
KMC: Kangaroo Mother Care
LSHTM: London School of Hygiene & Tropical Medicine
NEST360: Newborn Essential Solutions and Technologies
NID: Neonatal Inpatient Dataset
QED: Quality, Equity, Dignity
QI: Quality Improvement
SDGs: Sustainable Development Goal
SNCU: Special Newborn Care Units
SSNC: Small and Sick Newborn Care
TPN: Total Parenteral Nutrition

## References

[1] World Health Organization (WHO). Every Newborn Action Plan. https://www.who.int/initiatives/every-newborn-action-plan. Accessed 9 Aug 2022.

[2] World Health Organization (WHO). SDG Target 3.2: Newborn and child mortality. https://www.who.int/data/gho/data/themes/topics/indicator-groups/indicator-group-details/GHO/sdg-target-3.2-newborn-and-child-mortality. Accessed 9 Aug 2022.

[3] UN-IGME child mortality report: https://childmortality.org/wpcontent/uploads/2023/01/UN-IGME-Child-Mortality-Report-2022.pdf.

[4] UNICEF. Monitoring the situation of women and children. https://data.unicef.org/topic/maternal-health/delivery-care/. Accessed 9 Aug 2022.

[5] World Health Organisation (WHO). Monitoring the building blocks of health systems: a handbook of indicators and their measurement strategies. https://apps.who.int/iris/handle/10665/258734. Accessed 9 Aug 2022.

[6] Tuti T, Aluvaala J, Chelangat D, Mbevi G, Wainaina J, Mumelo L, et al. Improving in-patient neonatal data quality as a pre-requisite for monitoring and improving quality of care at scale: a multisite retrospective cohort study in Kenya. PLOS Glob Public Health. 2022;2:e0000673. 36962543 10.1371/journal.pgph.0000673 PMC10021237

[7] Lundin R, Mariani I, Peven K, Day LT, Lazzerini M. Quality of routine health facility data used for newborn indicators in low- and middle-income countries: A systematic review. J Glob Health. 2022;12(1):04019.

[8] Save the Children. Improving Availability and Quality of Routine Data for Newborns: Malawi’s experience with kangaroo mother care (KMC). https://resourcecentre.savethechildren.net/document/improving-availability-and-quality-routine-data-newborns-malawis-experience-kangaroo-mother/. Accessed 26 Aug 2022.

[9] DHIS2. https://dhis2.org/. Accessed 9 Aug 2022.

[10] Bhattacharya AA, Umar N, Audu A, Allen E, Schellenberg JRM, Marchant T. Quality of routine facility data for monitoring priority maternal and newborn indicators in DHIS2: a case study from Gombe State, Nigeria. PLoS One. 2019;14:e0211265. 30682130 10.1371/journal.pone.0211265 PMC6347394

[11] Rumisha SF, Lyimo EP, Mremi IR, Tungu PK, Mwingira VS, Mbata D, et al. Data quality of the routine health management information system at the primary healthcare facility and district levels in Tanzania. BMC Med Inform Decis Mak. 2020;20:1–22. 33334323 10.1186/s12911-020-01366-w PMC7745510

[12] Shamba D, Day LT, Zaman SB, Sunny AK, Tarimo MN, Peven K, et al. Barriers and enablers to routine register data collection for newborns and mothers: EN-BIRTH multi-country validation study. BMC Pregnancy Childbirth. 2021;21:1–14. 33765963 10.1186/s12884-020-03517-3 PMC7995573

[13] Salim N, Shabani J, Peven K, Rahman QS, Kc A, Shamba D, et al. Kangaroo mother care: EN-BIRTH multi-country validation study. BMC Pregnancy Childbirth. 2021;21:1–16. 33765950 10.1186/s12884-020-03423-8 PMC7995571

[14] Rahman AE, Hossain AT, Zaman SB, Salim N, Ashish KC, Day LT, et al. Antibiotic use for inpatient newborn care with suspected infection: EN-BIRTH multi-country validation study. BMC Pregnancy Childbirth. 2021;21:1–17. 33765948 10.1186/s12884-020-03424-7 PMC7995687

[15] Day LT, Sadeq-urRahman Q, EhsanurRahman A, Salim N, Ashish KC, Ruysen H, et al. Assessment of the validity of the measurement of newborn and maternal health-care coverage in hospitals (EN-BIRTH): an observational study. Lancet Glob Health. 2021;9:e267–79. 33333015 10.1016/S2214-109X(20)30504-0

[16] Vermont Oxford Network. https://public.vtoxford.org/. Accessed 16 Jan 2023.

[17] Horbar JD, Soll RF, Edwards WH. The Vermont Oxford Network: a community of practice. Clin Perinatol. 2010;37:29–47. 20363446 10.1016/j.clp.2010.01.003

[18] National Health Service. Data Model and Dictionary. Neonatal Critical Care Minimum Data Set. https://www.datadictionary.nhs.uk/data_sets/supporting_data_sets/neonatal_critical_care_minimum_data_set.html#dataset_neonatal_critical_care_minimum_data_set.overview. Accessed 16 Jan 2023.

[19] Eunice Kennedy Shriver National Institute of Child Health and Human Development. Neonatal Research Network. https://neonatal.rti.org/. Accessed 16 Jan 2023.

[20] Beltempo M, Shah PS, Lee SK. The Canadian Neonatal Network: development, evolution, and progress. Pediatr Med. 2021;0:0–0.

[21] National Health Mission - India, UNICEF. Best practices-facility based newborn care data base. https://www.nhmmp.gov.in/WebContent/BestPractice/Best%20Practices%20-%20Facility%20Based%20Newborn%20Care%20Data%20Base.pdf. Accessed 4 Apr 2023.

[22] Tuti T, Bitok M, Malla L, Paton C, Muinga N, Gathara D, et al. Improving documentation of clinical care within a clinical information network: an essential initial step in efforts to understand and improve care in Kenyan hospitals. BMJ Glob Health. 2016;1:e000028. 27398232 10.1136/bmjgh-2016-000028 PMC4934599

[23] World Health Organisation (WHO). Quality of Care Network. https://www.qualityofcarenetwork.org/index.php/about. Accessed 5 Dec 2022.

[24] NEST360. Information Tools. https://nest360.org/project/info-tools/. Accessed 5 Apr 2023.

[25] World Health Organisation (WHO). Standards for improving the quality of care for small and sick newborns in health facilities. https://www.who.int/publications/i/item/9789240010765. Accessed 9 Aug 2022.

[26] World Health Organisation (WHO). Data Principles. 2019. https://www.who.int/data/principles. Accessed 16 Aug 2022.

[27] Maina M, Aluvaala J, Mwaniki P, Tosas-Auguet O, Mutinda C, Maina B, et al. Using a common data platform to facilitate audit and feedback on the quality of hospital care provided to sick newborns in Kenya. BMJ Glob Health. 2018;3:e001027. 30258654 10.1136/bmjgh-2018-001027 PMC6150140

[28] Muinga N, Paton C, Gicheha E, Omoke S, Abejirinde IOO, Benova L, et al. Using a human-centred design approach to develop a comprehensive newborn monitoring chart for inpatient care in Kenya. BMC Health Serv Res. 2021;21:1–14. 34556098 10.1186/s12913-021-07030-x PMC8461871

[29] Carns J, Kawaza K, Liaghati-Mobarhan S, Asibon A, Quinn MK, Chalira A, et al. Neonatal CPAP for respiratory distress across Malawi and mortality. Pediatrics. 2019;144:668. 10.1542/peds.2019-0668 31540968

[30] Hagel C, Paton C, Mbevi G, English M. Data for tracking SDGs: challenges in capturing neonatal data from hospitals in Kenya. BMJ Glob Health. 2020;5:e002108. 32337080 10.1136/bmjgh-2019-002108 PMC7170465

[31] Vermont Oxford Network. Global Neonatal Database. https://public.vtoxford.org/data-and-reports/global-neonatal-database/. Accessed 9 Aug 2022.

[32] Liverpool School of Tropical Medicine. Neonatal Nutrition Network (NNN). https://www.lstmed.ac.uk/research/departments/clinical-sciences/neonatal-nutrition-network. Accessed 28 Sep 2022.

[33] Lawn JE, Mwansa-Kambafwile J, Horta BL, Barros FC, Cousens S. Immediate “Kangaroo Mother Care” and survival of infants with low birth weight. N Engl J Med. 2021;384:2028–38. 10.1056/NEJMoa2026486 PMC8108485 34038632

[34] Adejuyigbe EA, Anand P, Ansong D, Anyabolu CH, Arya S, Assenga E, et al. Impact of continuous Kangaroo Mother Care initiated immediately after birth (iKMC) on survival of newborns with birth weight between 1.0 to < 1.8 kg: study protocol for a randomized controlled trial. Trials. 2020;21:1–13. 32188485 10.1186/s13063-020-4101-1 PMC7081677

[35] International Severe Acute Respiratory and emerging Infections Consortium (ISARIC). COVID-19 Case Report Form. https://isaric.org/research/covid-19-clinical-research-resources/covid-19-crf/. Accessed 9 Aug 2022.

[36] Zhu H, Wang L, Fang C, Peng S, Zhang L, Chang G, et al. Clinical analysis of 10 neonates born to mothers with 2019-nCoV pneumonia. Transl Pediatr. 2020;9:510–60. 10.21037/tp.2020.02.06 PMC7036645 32154135

[37] Kirtsman M, Diambomba Y, Poutanen SM, Malinowski AK, Vlachodimitropoulou E, Parks WT, et al. Probable congenital sars-cov-2 infection in a neonate born to a woman with active sars-cov-2 infection. CMAJ. 2020;192:E647–50. 32409520 10.1503/cmaj.200821 PMC7828840

[38] Zeng L, Xia S, Yuan W, Yan K, Xiao F, Shao J, et al. Neonatal early-onset infection with SARS-CoV-2 in 33 neonates born to mothers with COVID-19 in Wuhan, China. JAMA Pediatr. 2020;174:722–5. 32215598 10.1001/jamapediatrics.2020.0878 PMC7099530

[39] Medvedev MM, Tumukunde V, Mambule I, Tann CJ, Waiswa P, Canter RR, et al. Operationalising kangaroo Mother care before stabilisation amongst low birth Weight Neonates in Africa (OMWaNA): Protocol for a randomised controlled trial to examine mortality impact in Uganda. Trials. 2020;21:1–19. 32005286 10.1186/s13063-019-4044-6 PMC6995072

[40] Fajolu IB, Mairami AB, Okonkwo I, Ezenwa B, Otuneye AT, Amuabunos EA, et al. Rates and predictors of mortality of very low birthweight infants in three Nigerian tertiary hospitals. Acta Paediatr. 2022;111(1):67–74. 10.1111/apa.16554 36170565

[41] Chang W, Cheng J, Allaire J, Sievert C, Schloerke B, Xie Y, et al. shiny: Web Application Framework for R. R package version 1.7.4.9001. 2023. https://shiny.rstudio.com/. Accessed 10 Jan 2023.

[42] Harris PA, Taylor R, Thielke R, Payne J, Gonzalez N, Conde JG. Research electronic data capture (REDCap)—a metadata-driven methodology and workflow process for providing translational research informatics support. J Biomed Inform. 2009;42:377–81. 18929686 10.1016/j.jbi.2008.08.010 PMC2700030

[43] Lawn JE, Blencowe H, Oza S, You D, Lee ACC, Waiswa P, et al. Every newborn: progress, priorities, and potential beyond survival. The Lancet. 2014;384:189–205. 10.1016/S0140-6736(14)60496-7 24853593

[44] Lawn JE, Cousens S, Zupan J. 4 million neonatal deaths: when? Where? Why? Lancet. 2005;365:891–900. 15752534 10.1016/S0140-6736(05)71048-5

[45] UNICEF. Global Development Commons. 2019 India: Special Newborn Care Units to help reduce newborn deaths in India 2007–2017. https://gdc.unicef.org/resource/2019-india-special-newborn-care-units-help-reduce-newborn-deaths-india-2007-2017. Accessed 16 Aug 2022.

[46] National Health Mission - India. SNCU Online data management and follow up tracking system. 2011. https://www.nhm.gov.in/images/pdf/nrhm-updates/2nd_child_health/pilot_imp_sncu_software_dr_gagan.pdf. Accessed 4 Apr 2023.

[47] National Health Mission - India, UNICEF. Facility Based Newborn Care Database. https://sncuindiaonline.org/d_loginAction. Accessed 5 Apr 2023.

[48] World Health Organisation (WHO). Survive and Thrive: Transforming care for every small and sick newborn; 2019. https://www.who.int/publications/i/item/9789241515887. Accessed 11 Jan 2023.

[49] World Health Organisation (WHO). Task shifting: rational redistribution of tasks among health workforce teams : global recommendations and guidelines. https://apps.who.int/iris/handle/10665/43821. Accessed 11 Jan 2023.

[50] Bohne C, Zimba E, Lufesi N, Asibon A, Salim N, Masanja H, et al. Theory of Change to Accelerate Scale Up of High-Quality Small and Sick Newborn Care in Kenya, Malawi, Nigeria, and Tanzania with NEST360. BMC. 2023.

[51] Shah A. Using data for improvement. BMJ. 2019;364:l189. 30770353 10.1136/bmj.l189 PMC6376414

[52] Goodwin S. Data rich, information poor (DRIP) syndrome: is there a treatment? Radiol Manage. 1996;12:45–9. 10158370

[53] NEST360. https://nest360.org/. Accessed 9 Nov 2023.

[54] Day LT, Ruysen H, Gordeev VS, Gore-Langton GR, Boggs D, Cousens S, et al. “Every Newborn-BIRTH” protocol: observational study validating indicators for coverage and quality of maternal and newborn health care in Bangladesh, Nepal and Tanzania. J Glob Health. 2019;9:010902. 30863542 10.7189/jogh.09.01902 PMC6406050

[55] Moxon SG, Ruysen H, Kerber KJ, Amouzou A, Fournier S, Grove J, et al. Count every newborn; a measurement improvement roadmap for coverage data. BMC Pregnancy Childbirth. 2015;15:1–23. 26391444 10.1186/1471-2393-15-S2-S8 PMC4577758

[56] Stevenson AG, Tooke L, Edwards EM, Mangiza M, Horn D, Heys M, et al. The use of data in resource limited settings to improve quality of care. Semin Fetal Neonatal Med. 2021;26:101204. 33579628 10.1016/j.siny.2021.101204

[57] Heywood A, Rohde J. Using information for action - a manual for health workers at facility level. https://pdf.usaid.gov/pdf_docs/Pnacx654.pdf. Accessed 8 Nov 2023.

[58] MEASURE Evaluation. Defining Electronic Health Technologies and Their Benefits for Global Health Program Managers: Data Interoperability and Data Integration. 2015. https://www.measureevaluation.org/resources/publications/fs-15-165f.html. Accessed 16 Aug 2022.

[59] Braa J, Sahey S. Integrated health information architecture - power to the users: design, development and use. Matrix publishers. 2012.

[60] Slattery P, Saeri AK, Bragge P. Research co-design in health: a rapid overview of reviews. Health Res Policy Syst. 2020;18:1–13. 32046728 10.1186/s12961-020-0528-9 PMC7014755

[61] Nothacker M, Stokes T, Shaw B, Lindsay P, Sipilä R, Follmann M, et al. Reporting standards for guideline-based performance measures. Implement Sci. 2016;11:1–11. 26772173 10.1186/s13012-015-0369-z PMC4714427

[62] Lazzerini M, Senanayake H, Mohamed R, et al. Implementation of an individual patient prospective database of hospital births in Sri Lanka and its use for improving quality of care. BMJ Open. 2019;9:e023706. 10.1136/bmjopen-2018-023706 PMC6368149 30782885

[63] Seale AC, Barsosio HC, Koech AC, Berkley JA. Embedding surveillance into clinical care to detect serious adverse events in pregnancy. Vaccine. 2015;33:6466. 26254977 10.1016/j.vaccine.2015.07.086 PMC4817214

[64] Bergsjø P, Mlay J, Lie RT, Lie-Nielsen E, Shao JF. A medical birth registry at Kilimanjaro Christian Medical Centre. East Afr J Public Health. 2007;4:1–4. 17907753

[65] Sørbye IK, Vangen S, Oneko O, Sundby J, Bergsjø P. Caesarean section among referred and self-referred birthing women: a cohort study from a tertiary hospital, northeastern Tanzania. BMC Pregnancy Childbirth. 2011;11:1–10. 21798016 10.1186/1471-2393-11-55 PMC3160415

[66] Save the Children. Newborn Health in Humanitarian Settings Field Guide. https://resourcecentre.savethechildren.net/document/newborn-health-humanitarian-settings-field-guide/. Accessed 15 Aug 2022.

[67] Lawn JE, Davidge R, Paul VK, Von XS, De Graft JJ, Costello A, et al. Born Too Soon: care for the preterm baby. Reprod Health. 2013;10(SUPPL. 1):1–19. 24625233 10.1186/1742-4755-10-S1-S5 PMC3828583

[68] Seale AC, Blencowe H, Zaidi A, Ganatra H, Syed S, Engmann C, et al. Neonatal severe bacterial infection impairment estimates in South Asia, sub-Saharan Africa, and Latin America for 2010. Pediatr Res. 2013;74:73. 24366464 10.1038/pr.2013.207 PMC3873707

[69] Bhutani VK, Zipursky A, Blencowe H, Khanna R, Sgro M, Ebbesen F, et al. Neonatal hyperbilirubinemia and Rhesus disease of the newborn: incidence and impairment estimates for 2010 at regional and global levels. Pediatr Res. 2013;74:86–100. 24366465 10.1038/pr.2013.208 PMC3873706

[70] Blencowe H, Lawn JE, Vazquez T, Fielder A, Gilbert C. Preterm-associated visual impairment and estimates of retinopathy of prematurity at regional and global levels for 2010. Pediatr Res. 2013;74:35–49. 24366462 10.1038/pr.2013.205 PMC3873709

[71] NEST360/UNICEF. Small and Sick Newborn Care Implementation Toolkit. https://www.newborntoolkit.org/. Accessed 16 Jan 2023.

