# Supplementary material for: Neonatal inpatient dataset for small and sick newborn care in low- and middle-income countries: systematic development and multi-country operationalisation with NEST360
Source: BMC Pediatr. 2023 Nov 15;23(Suppl 2):567. doi: 10.1186/s12887-023-04341-2 (PMC10652643; doi:10.1186/s12887-023-04341-2)
Supplement: Supplementary file 1 — Additional file 1. WHO Levels of Newborn Care with Interventions - NEST360 Informing the ‘How To’ for Implementation. [file 12887_2023_4341_MOESM1_ESM.pdf]

WHO Levels of Newborn Care with Interventions NEST360 Informing the 'How To' for Implementation

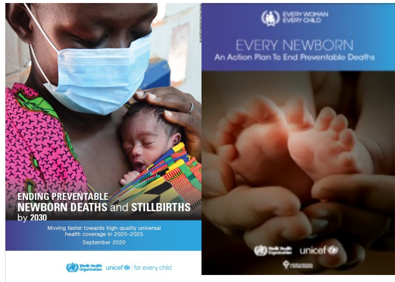

**Level 1**<br>**Immediate and Essential Newborn Care**	Immediate newborn care (delayed cord clamping, drying, skin-to-skin etc.)<br>Neonatal resuscitation for those who need it<br>Breastfeeding early initiation and support<br>Essential newborn care Identification and referral of complications<br>Targeted care as needed e.g., PMTCT of HIV
**Level 2**<br>**Special Newborn Care**	**Thermal care including KMC for all stable neonates <2000gms**<br>**Assisted feeding and IV fluids**<br>**Safe administration of oxygen**<br>**Sepsis detection and management of with injection antibiotics**<br>**Jaundice detection and management of with phototherapy**<br>**Neonatal encephalopathy detection and management**<br>**Congenital abnormalities detection and referral/management**
**Transition to Intensive Care (2+)**	**CPAP management of preterm respiratory distress**<br>Follow up of at-risk newborns<br>Exchange transfusion
**Level 3**<br>**Intensive Newborn Care**	Mechanical/assisted ventilation<br>Advanced feeding support (e.g., parenteral nutrition)<br>Paediatric surgery for congenital conditions<br>Screening and treatment for RoP

NEST360°

EVERY NEWBORN COVERAGE TARGETS 2020-2025

80% of districts have at least one level-2 in-patient unit to care for small & sick newborns, with respiratory support including CPAP.

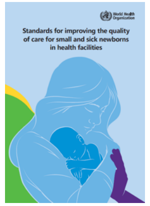

Ref: WHO/UNICEF report "Survive and Thrive; Transforming care for small and sick newborns" based on a global survey, PLoS (Moxon et al 2019)

WHO norms and stds for SSNC and in ENAP coverage targets .
