# Supplementary material for: Neonatal inpatient dataset for small and sick newborn care in low- and middle-income countries: systematic development and multi-country operationalisation with NEST360
Source: BMC Pediatr. 2023 Nov 15;23(Suppl 2):567. doi: 10.1186/s12887-023-04341-2 (PMC10652643; doi:10.1186/s12887-023-04341-2)
Supplement: Supplementary file 2 — Additional file 2. Summary list of Nairobi 2019 workshop participants by characteristic. [file 12887_2023_4341_MOESM2_ESM.pdf]

SUPPLEMENTAL INFORMATION – ADDITIONAL FILE 2

SUPPLEMENT TITLE

Small and sick newborn care: African-led implementation research

PAPER TITLE

Neonatal inpatient dataset for small and sick newborn care in low- and middle-income countries: systematic development and multi-country operationalisation with NEST360.

Draft for Discussion

Additional File 2: *Summary list of Nairobi 2019 workshop participants by characteristic*

Participants (n=18)	Number (%)
Clinical Background	11 (61%)
Clinician	9 (50%)
Nurse	2 (11%)
Government Policymaker	6 (33%)
Academic	14 (78%)
