# Supplementary material for: Neonatal inpatient dataset for small and sick newborn care in low- and middle-income countries: systematic development and multi-country operationalisation with NEST360
Source: BMC Pediatr. 2023 Nov 15;23(Suppl 2):567. doi: 10.1186/s12887-023-04341-2 (PMC10652643; doi:10.1186/s12887-023-04341-2)
Supplement: Supplementary file 3 — Additional file 3. Neonatal Inpatient Dataset paper tool. [file 12887_2023_4341_MOESM3_ESM.pdf]

NEST360°

Neonatal Inpatient Form - Final Dataset

Facility Name [ ]

Record ID [ ][ ][ ] - [ ][ ][ ][ ][ ]

Date Y Y Y Y M M D D

AFFIX RECORD ID

Abstractor ID [ ][ ]

Abstractor Name [ ]

Position: Data Clerk [ ] Data Officer [ ] Nurse [ ] If other - specify? [ ]
Nurse-in-charge [ ] Medical Doctor [ ] Other [ ]

Admission Information

Baby: First Name [ ] Last Name [ ]

Baby Hospital ID [ ] Not recorded / Not readable — Registration No. [ ] Not recorded / Not readable

Mother: First Name [ ] Last Name [ ]

Readmission: Yes / No / Not recorded / Not readable

How many phone numbers are recorded for the mother or family? 0 [ ] 1 [ ] 2 [ ]

Phone Number(s) 1) 2) Not recorded / Not readable

Patient's Residence:

State:

Abia [ ]	Adamawa [ ]	Akwa Ibom [ ]	Anambra [ ]	Bauchi [ ]	Bayelsa [ ]	Benue [ ]
Borno [ ]	Cross River [ ]	Delta [ ]	Ebonyi [ ]	Edo [ ]	Ekiti [ ]	Enugu [ ]
Gombe [ ]	Imo [ ]	Jigawa [ ]	Kaduna [ ]	Kano [ ]	Katsina [ ]	Kebbi [ ]
Kogi [ ]	Kwara [ ]	Lagos [ ]	Nasarawa [ ]	Niger [ ]	Ogun [ ]	Ondo [ ]
Osun [ ]	Oyo [ ]	Plateau [ ]	Rivers [ ]	Sokoto [ ]	Taraba [ ] Yobe [ ]	Zamfara [ ]

Local Government Area [ ] Ward [ ]

Address Notes/Directions [ ]

Date of Birth Y Y Y Y M M D D — Not recorded / Not readable
Time of Birth H H M M — Not recorded / Not readable

Date of Admission Y Y Y Y M M D D — Not recorded / Not readable
Time of Admission H H M M — Not recorded / Not readable

Birth Location: Inborn [ ] Outborn [ ] Not recorded / Not readable

If outborn - where? Home [ ] In Transit [ ] Religious Building [ ] Other Healthcare Facility [ ] Other (i.e. Not Healthcare Facility) [ ] Not recorded / Not readable

If other healthcare facility:

- Healthcare Centre, Dispensary or Other?
  Hospital [ ] Healthcare Centre [ ] Dispensary [ ]
  Traditional Birth Attendant (TBA) Facility [ ]
  Maternity Home [ ] Other [ ] Not recorded / Not readable
- Private, Public, Parastatal or Faith-Based: Public [ ] Private [ ]
  Parastatal [ ] Faith-Based [ ] Not recorded / Not readable
- Name [ ]

If other (i.e. not a healthcare facility):

- Enter a location [ ]

Reason for referral from facility

Blood transfusion/exchange [ ] Specialist review (Cardiac, Neurology etc.) [ ] Mechanical ventilation [ ]
Neonatal intensive care [ ] Dialysis [ ] Surgery/Surgical management [ ]
Mother request - nearest facility [ ] Mother/Family request [ ] Further management (not explained) [ ]
Mother transferred for further management [ ] Other reason [ ] Not recorded / Not readable

Other reason - specify [ ]

Weight on admission (grams) [ ][ ][ ][ ] g Not recorded / Not readable

Neonatal Inpatient Form - Final Dataset

NEST360°

Abstractor Name [ ] Record ID [| |] - [| | | |] Date Y Y Y Y M M D D

Reason for Admission

Reason for Admission (select one per column)

	First (1)	Second (2)	Third (3)
1. Congenital Malformation	☐	☐	☐
2. Small Baby / Preterm / Low Birthweight	☐	☐	☐
3. "Birth asphyxia" / Birth Injury	☐	☐	☐
4. Suspected infection - sepsis/meningitis	☐	☐	☐
5. Tetanus	☐	☐	☐
6. Respiratory Distress / Difficulty in Breathing	☐	☐	☐
7. Convulsions	☐	☐	☐
8. Temperature too high	☐	☐	☐
9. Temperature too low	☐	☐	☐
10. Jaundice	☐	☐	☐
11. Vomiting	☐	☐	☐
12. Pallor / Severe Anaemia	☐	☐	☐
13. Cord red / sticky	☐	☐	☐
14. Not feeding / Failure to suck	☐	☐	☐
15. Large Baby	☐	☐	☐
16. PROM and Maternal Fever	☐	☐	☐
17. Other maternal reasons	☐	☐	☐
18. Other (specify)	☐	☐	☐
	Not recorded	Not recorded	Not recorded
	Not readable	Not readable	Not readable

If other - specify (First) [ ]

If other - specify (Second) [ ]

If other - specify (Third) [ ]

Maternal and Birth Details

Maternal DOB: Day D D Not recorded Not readable Month M M Not recorded Not readable Year Y Y Y Y Not recorded Not readable

Maternal Age (Years) [|] Not recorded Not readable Mother Alive Yes No Not recorded Not readable

Maternal Cause of Death Embolism ☐ Haemorrhage ☐ Hypertension ☐ Sepsis ☐
Other direct causes (e.g. complications of delivery, obstructed labour and other) ☐
Other indirect causes (e.g. HIV-related, pre-existing medical conditions and other) ☐ Not recorded Not readable

Delivery Type Singleton ☐ Twin ☐ Triplet ☐ Quad ☐ Other multiple ☐ Conjoined twin ☐ Not recorded Not readable

Other multiple - specify [|]

If multiple - order this newborn was delivered
1st ☐ 2nd ☐ 3rd ☐ 4th ☐ 5th ☐ 6th ☐ 7th ☐ 8th ☐ Not recorded Not readable

Sibling #1

Medical state - sibling (1)
Alive ☐ Dead ☐ Not recorded Not readable

If sibling (1) died:
Dead before Admission ☐ Dead after Admission - at this facility ☐
Dead after Admission - NOT at this facility ☐ Not recorded Not readable

Was sibling (1) admitted? No ☐ Admitted at this facility ☐ Admitted at another facility ☐ Not recorded Not readable

Sibling (1) - NEST360° Record No. [ ] Not recorded Not readable
Sibling (1) - Baby Hospital ID [ ] Not recorded Not readable
Sibling (1) - Registration No. [ ] Not recorded Not readable

Sibling #2

Medical state - sibling (2)
Alive ☐ Dead ☐ Not recorded Not readable

If sibling (2) died:
Dead before Admission ☐ Dead after Admission - at this facility ☐
Dead after Admission - NOT at this facility ☐ Not recorded Not readable

Was sibling (2) admitted? No ☐ Admitted at this facility ☐ Admitted at another facility ☐ Not recorded Not readable

Sibling (2) - NEST360° Record No. [ ] Not recorded Not readable
Sibling (2) - Baby Hospital ID [ ] Not recorded Not readable
Sibling (2) - Registration No. [ ] Not recorded Not readable

Neonatal Inpatient Form - Final Dataset

Abstractor Name [ ] Record ID [ ][ ][ ] - [ ][ ][ ][ ][ ] Date Y Y Y Y M M D D

Maternal and Birth Details (cont.)

Sibling #3

Medical state - sibling (3)
Alive [ ] Dead [ ] Not recorded Not readable

If sibling (3) died:
Dead before Admission [ ] Dead after Admission - at this facility [ ]
Dead after Admission - NOT at this facility [ ] Not recorded Not readable

Was sibling (3) admitted? No [ ] Admitted at this facility [ ] Admitted at another facility [ ] Not recorded Not readable

Sibling (3) - NEST360° Record No. [ ] Not recorded Not readable
Sibling (3) - Baby Hospital ID [ ] Not recorded Not readable
Sibling (3) - Registration No. [ ] Not recorded Not readable

Sibling #4

Medical state - sibling (4)
Alive [ ] Dead [ ] Not recorded Not readable

If sibling (4) died:
Dead before Admission [ ] Dead after Admission - at this facility [ ]
Dead after Admission - NOT at this facility [ ] Not recorded Not readable

Was sibling (4) admitted? No [ ] Admitted at this facility [ ] Admitted at another facility [ ] Not recorded Not readable

Sibling (4) - NEST360° Record No. [ ] Not recorded Not readable
Sibling (4) - Baby Hospital ID [ ] Not recorded Not readable
Sibling (4) - Registration No. [ ] Not recorded Not readable

Sibling #5

Medical state - sibling (5)
Alive [ ] Dead [ ] Not recorded Not readable

If sibling (5) died:
Dead before Admission [ ] Dead after Admission - at this facility [ ]
Dead after Admission - NOT at this facility [ ] Not recorded Not readable

Was sibling (5) admitted? No [ ] Admitted at this facility [ ] Admitted at another facility [ ] Not recorded Not readable

Sibling (5) - NEST360° Record No. [ ] Not recorded Not readable
Sibling (5) - Baby Hospital ID [ ] Not recorded Not readable
Sibling (5) - Registration No. [ ] Not recorded Not readable

Sibling #6

Medical state - sibling (6)
Alive [ ] Dead [ ] Not recorded Not readable

If sibling (6) died:
Dead before Admission [ ] Dead after Admission - at this facility [ ]
Dead after Admission - NOT at this facility [ ] Not recorded Not readable

Was sibling (6) admitted? No [ ] Admitted at this facility [ ] Admitted at another facility [ ] Not recorded Not readable

Sibling (6) - NEST360° Record No. [ ] Not recorded Not readable
Sibling (6) - Baby Hospital ID [ ] Not recorded Not readable
Sibling (6) - Registration No. [ ] Not recorded Not readable

Sibling #7

Medical state - sibling (7)
Alive [ ] Dead [ ] Not recorded Not readable

If sibling (7) died:
Dead before Admission [ ] Dead after Admission - at this facility [ ]
Dead after Admission - NOT at this facility [ ] Not recorded Not readable

Was sibling (7) admitted? No [ ] Admitted at this facility [ ] Admitted at another facility [ ] Not recorded Not readable

Sibling (7) - NEST360° Record No. [ ] Not recorded Not readable
Sibling (7) - Baby Hospital ID [ ] Not recorded Not readable
Sibling (7) - Registration No. [ ] Not recorded Not readable

Mode of Delivery
Normal vertex [ ] Forceps [ ] Vacuum extraction [ ] Vaginal breech [ ] C-section [ ] Not recorded Not readable

Antenatal Corticosteroids: Yes No Not recorded Not readable

Maternal HIV status: Negative [ ] Unknown [ ] Positive [ ] Not recorded Not readable

HIV Treatment - Mother: Yes No Not recorded Not readable
HIV Treatment - Baby: Yes No Not recorded Not readable

Maternal Chronic Conditions: Diabetes [ ] Hypertension [ ] Other [ ] None [ ] Not recorded Not readable

Type of Diabetes: Pregnancy-induced [ ] Pre-existing [ ] Not recorded Not readable

Pre-Existing Diabetes: Type 1 [ ] Type 2 [ ] Not recorded Not readable

Type of Hypertension: Pregnancy-induced [ ] Pre-existing [ ] Not recorded Not readable

If other chronic condition - specify [ ]

Neonatal Inpatient Form - Final Dataset

Abstractor Name [ ] Record ID [| |] - [| | | |] Date Y Y Y Y M M D D

SARS-CoV-2 (COVID-19)

Has the mother been tested for SARS-CoV-2 (COVID-19) in the last 14 days? Yes No Not recorded Not readable

Mother SARS-Cov-2 Test (COVID-19) Result

Test Negative ☐ Test Positive ☐ Test Done but Unknown Result ☐ Not recorded Not readable

Date of Sample Collection (Mother) Y Y Y Y M M D D Not recorded Not readable

Has the mother been presumed infected with SARS-CoV-2 (COVID-19) based on signs and symptoms in the last 14 days? Yes No Not recorded Not readable

Were the mother's SARS-CoV-2 (COVID-19) signs and symptoms mild, moderate or Mild ☐ Moderate ☐ Severe ☐ Not recorded Not readable

In the last 14 days, has the mother...

Had a fever (≥37.5°)?	Yes	No	Not recorded	Not readable
Lost her sense of smell and/or taste?	Yes	No	Not recorded	Not readable
Had mild or moderate respiratory symptoms?	Yes	No	Not recorded	Not readable
Suffered from respiratory failure?	Yes	No	Not recorded	Not readable
Suffered from septic shock or multiple organ failure?	Yes	No	Not recorded	Not readable

Has the newborn been tested for SARS-CoV-2 (COVID-19) since birth? Yes No Not recorded Not readable

Newborn SARS-CoV-2 Test (COVID-19) Result

Test Negative ☐ Test Positive ☐ Test Done but Unknown Result ☐ Not recorded Not readable

Date of Sample Collection (Newborn) Y Y Y Y M M D D

Was the newborn tested for SARS-CoV-2 (COVID-19) because the mother tested positive or due to maternal signs and symptoms? Yes No Not recorded Not readable

Infant Details

Birth Weight (grams) [| | |] g Not recorded Not readable

How is gestational age documented? Exact number of Months, Weeks or Days ☐

Preterm ☐ Early term ☐ Full term ☐ Late term ☐ Post-term ☐ Not recorded Not readable

What format is the gestational age data recorded in? Number of Weeks only ☐ Number of Months only ☐

Number of Months and Weeks ☐ Number of Months, Weeks and Days ☐ Not recorded Not readable

Months: [|] Weeks: [|] Days: [ ]

Method used to measure gestational age Early ultrasound (1st Trimester) ☐ Late ultrasound (2nd or 3rd Trimester ☐

Postnatal clinical gestational assessment ☐ Fundal height ☐ LMP ☐ Not recorded Not readable

If postnatal clinical gestational assessment - which method?

Ballard Method ☐ Dubowitz Method ☐ Finnstrom Method ☐ Other ☐ Not recorded Not readable

If other - what method? [ ]

Newborn Sex Male ☐ Female ☐ Indeterminate ☐ Not recorded Not readable

Observations and Nursing Care

Was temperature recorded on admission? Yes No Not recorded Not readable

In what units was temperature measured? °C ☐ °F ☐ Not recorded Not readable

Temperature on admission [|] . [ ]

Oxygen saturation (%) on admission [|] % Not recorded Not readable

Signs of severe respiratory distress (e.g. severe in-drawing, grunting, high respiratory rate)	Yes	No	Not recorded	Not readable
Cord Chlorhexidine Administered	Yes	No	Not recorded	Not readable
Vitamin K Administered	Yes	No	Not recorded	Not readable
Was blood sugar recorded on admission?	Yes	No	Not recorded	Not readable

In what units was blood sugar measured? mmol/L ☐ mg/dL ☐ Not recorded Not readable

Blood sugar concentration on admission [|] . [ ]

Neonatal Inpatient Form - Final Dataset

Abstractor Name ______ Record ID ___ - _____ Date Y Y Y Y M M D D

Microbiology and Laboratory

Blood culture for suspected sepsis — Not Done ☐ Done - Culture Negative ☐ Done - Culture Positive ☐ Done - Unknown Result ☐ Not readable

Culture Positive Organism **(Blood)**: Staphylococcus aureus ☐ Klebsiella spp. ☐ Pseudomonas spp. ☐ Escherichia coli ☐ Acinetobacter spp. ☐ Group B Streptococcus ☐ Other gram-negatives ☐ Other Streptococcus ☐ Other Fungal spp. ☐ Other ☐ Not indicated ☐ Not recorded Not readable

If other or spp. - full species / genus ______

If culture positive - was antibiotic susceptibility testing done — Yes No Not recorded Not readable

Antibiotic Susceptibility Testing **(Blood)**	Not Done	Resistant	Intermediate	Sensitive		
Benzathine Penicillin	☐	☐	☐	☐	Not recorded	Not readable
Crystalline Penicillin	☐	☐	☐	☐	Not recorded	Not readable
Gentamicin	☐	☐	☐	☐	Not recorded	Not readable
Amikacin	☐	☐	☐	☐	Not recorded	Not readable
Ceftriaxone	☐	☐	☐	☐	Not recorded	Not readable
Ceftazidime	☐	☐	☐	☐	Not recorded	Not readable
Cefotaxime	☐	☐	☐	☐	Not recorded	Not readable
Piperazine	☐	☐	☐	☐	Not recorded	Not readable
Metronidazole	☐	☐	☐	☐	Not recorded	Not readable
Vancomycin	☐	☐	☐	☐	Not recorded	Not readable
Clindamycin	☐	☐	☐	☐	Not recorded	Not readable
Ampicillin	☐	☐	☐	☐	Not recorded	Not readable
Amoxycillin	☐	☐	☐	☐	Not recorded	Not readable
Flucloxacillin	☐	☐	☐	☐	Not recorded	Not readable
Meropenem	☐	☐	☐	☐	Not recorded	Not readable
Levofloxacin	☐	☐	☐	☐	Not recorded	Not readable
Other ______	☐	☐	☐	☐	Not recorded	Not readable
Other ______	☐	☐	☐	☐	Not recorded	Not readable
Other ______	☐	☐	☐	☐	Not recorded	Not readable
Other ______	☐	☐	☐	☐	Not recorded	Not readable
Other ______	☐	☐	☐	☐	Not recorded	Not readable

Were multiple microorganisms cultured from the same blood culture sample?
Yes - Identified ☐ Yes - Unidentified ☐ No ☐ Not recorded Not readable

If yes - specify full name of other isolated microorganism(s) ______

CSF culture for suspected meningitis — Not Done ☐ Done - Culture Negative ☐ Done - Culture Positive ☐ Done but Unknown Result ☐ Not readable

Culture Positive Organism **(CSF)**: Staphylococcus aureus ☐ Klebsiella spp. ☐ Pseudomonas spp. ☐ Escherichia coli ☐ Acinetobacter spp. ☐ Group B Streptococcus ☐ Other gram-negatives ☐ Other Streptococcus ☐ Other Fungal spp. ☐ Other ☐ Not indicated ☐ Not recorded Not readable

If other or spp. - full species / genus ______

If culture positive - was antibiotic susceptibility testing done — Yes No Not recorded Not readable

Antibiotic Susceptibility Testing **(CSF)**	Not Done	Resistant	Intermediate	Sensitive		
Benzathine Penicillin	☐	☐	☐	☐	Not recorded	Not readable
Crystalline Penicillin	☐	☐	☐	☐	Not recorded	Not readable
Gentamicin	☐	☐	☐	☐	Not recorded	Not readable
Amikacin	☐	☐	☐	☐	Not recorded	Not readable
Ceftriaxone	☐	☐	☐	☐	Not recorded	Not readable
Ceftazidime	☐	☐	☐	☐	Not recorded	Not readable
Cefotaxime	☐	☐	☐	☐	Not recorded	Not readable
Piperazine	☐	☐	☐	☐	Not recorded	Not readable
Metronidazole	☐	☐	☐	☐	Not recorded	Not readable
Vancomycin	☐	☐	☐	☐	Not recorded	Not readable
Clindamycin	☐	☐	☐	☐	Not recorded	Not readable
Ampicillin	☐	☐	☐	☐	Not recorded	Not readable
Amoxycillin	☐	☐	☐	☐	Not recorded	Not readable
Flucloxacillin	☐	☐	☐	☐	Not recorded	Not readable
Meropenem	☐	☐	☐	☐	Not recorded	Not readable
Levofloxacin	☐	☐	☐	☐	Not recorded	Not readable

Neonatal Inpatient Form - Final Dataset

Abstractor Name ☐ Record ID ☐☐☐ - ☐☐☐☐☐ Date Y Y Y Y M M D D

Microbiology and Laboratory (cont.)

Antibiotic Susceptibility Testing (**CSF**) (cont.)	Not Done	Resistant	Intermediate	Sensitive		
Other ☐	☐	☐	☐	☐	Not recorded	Not readable
Other ☐	☐	☐	☐	☐	Not recorded	Not readable
Other ☐	☐	☐	☐	☐	Not recorded	Not readable
Other ☐	☐	☐	☐	☐	Not recorded	Not readable
Other ☐	☐	☐	☐	☐	Not recorded	Not readable

Were multiple microorganisms cultured from the same CSF culture sample?

Yes - Identified ☐ Yes - Unidentified ☐ No ☐ Not recorded Not readable

If yes - specify full name of other isolated microorganism(s) ☐

Interventions

KMC Administered Yes No Not recorded Not readable

If yes - KMC Start Date Y Y Y Y M M D D Not recorded Not readable

If yes - KMC End Date Y Y Y Y M M D D Not recorded Not readable

Type of KMC practised Continuous ☐ Intermittent ☐ Sporadic ☐ Not recorded Not readable

Oxygen Therapy Administered Yes No Not recorded Not readable

How many times did the newborn receive oxygen therapy? ☐☐

Oxygen Therapy #1

Oxygen saturation (%) level that led to the use of oxygen therapy (1) ☐☐☐ % Not recorded Not readable

Oxygen therapy (1) Start Date Y Y Y Y M M D D Not recorded Not readable

Oxygen therapy (1) Start Time H H M M Not recorded Not readable

Oxygen therapy (1) End Date Y Y Y Y M M D D Not recorded Not readable

Oxygen therapy (1) End Time H H M M Not recorded Not readable

Oxygen Therapy #2

Oxygen saturation (%) level that led to the use of oxygen therapy (2) ☐☐☐ % Not recorded Not readable

Oxygen therapy (2) Start Date Y Y Y Y M M D D Not recorded Not readable

Oxygen therapy (2) Start Time H H M M Not recorded Not readable

Oxygen therapy (2) End Date Y Y Y Y M M D D Not recorded Not readable

Oxygen therapy (2) End Time H H M M Not recorded Not readable

Oxygen Therapy #3

Oxygen saturation (%) level that led to the use of oxygen therapy (3) ☐☐☐ % Not recorded Not readable

Oxygen therapy (3) Start Date Y Y Y Y M M D D Not recorded Not readable

Oxygen therapy (3) Start Time H H M M Not recorded Not readable

Oxygen therapy (3) End Date Y Y Y Y M M D D Not recorded Not readable

Oxygen therapy (3) End Time H H M M Not recorded Not readable

CPAP Administered Yes No Not recorded Not readable

If yes - How many times did the newborn receive CPAP? ☐☐

CPAP #1

Oxygen saturation (%) level that led to the use of CPAP (1) ☐☐☐ % Not recorded Not readable

CPAP (1) Start Date Y Y Y Y M M D D Not recorded Not readable

CPAP (1) Start Time H H M M Not recorded Not readable

CPAP (1) End Date Y Y Y Y M M D D Not recorded Not readable

CPAP (1) End Time H H M M Not recorded Not readable

Why was CPAP (1) stopped? No longer indicated (Improvement) ☐ No longer indicated (Deterioration) ☐

Power outage ☐ Device broke or stopped functioning ☐ Device needed for another baby ☐ Not recorded Not readable

CPAP #2

Oxygen saturation (%) level that led to the use of CPAP (2) ☐☐☐ % Not recorded Not readable

CPAP (2) Start Date Y Y Y Y M M D D Not recorded Not readable

CPAP (2) Start Time H H M M Not recorded Not readable

CPAP (2) End Date Y Y Y Y M M D D Not recorded Not readable

CPAP (2) End Time H H M M Not recorded Not readable

Neonatal Inpatient Form - Final Dataset

Abstractor Name [ ] Record ID [ ][ ][ ] - [ ][ ][ ][ ][ ] Date Y Y Y Y M M D D

Interventions (cont.)

CPAP #2 (cont.)

Why was CPAP (2) stopped? No longer indicated (Improvement) ☐ No longer indicated (Deterioration) ☐
Power outage ☐ Device broke or stopped functioning ☐ Device needed for another baby ☐ Not recorded | Not readable

CPAP #3

Oxygen saturation (%) level that led to the use of CPAP (3) [ ][ ][ ] % Not recorded | Not readable

CPAP (3) Start Date Y Y Y Y M M D D Not recorded | Not readable — CPAP (3) Start Time H H M M Not recorded | Not readable

CPAP (3) End Date Y Y Y Y M M D D Not recorded | Not readable — CPAP (3) End Time H H M M Not recorded | Not readable

Why was CPAP (3) stopped? No longer indicated (Improvement) ☐ No longer indicated (Deterioration) ☐
Power outage ☐ Device broke or stopped functioning ☐ Device needed for another baby ☐ Not recorded | Not readable

Did the infant receive mechanical ventilation? Yes | No | Not recorded | Not readable

How many times was the patient put on/off mechanical ventilation?
Once ☐ Twice ☐ Three times ☐ More than three times ☐ Not recorded | Not readable

Mechanical Ventilation #1

Oxygen saturation (%) level that led to the use of mechanical ventilation (1) [ ][ ][ ] % Not recorded | Not readable

Mech. ventilation (1) Start Date Y Y Y Y M M D D Not recorded | Not readable — Mech. ventilation (1) Start Time H H M M Not recorded | Not readable

Mech. ventilation (1) End Date Y Y Y Y M M D D Not recorded | Not readable — Mech, ventilation (1) End Time H H M M Not recorded | Not readable

Mechanical Ventilation #2

Oxygen saturation (%) level that led to the use of mechanical ventilation (2) [ ][ ][ ] % Not recorded | Not readable

Mech. ventilation (2) Start Date Y Y Y Y M M D D Not recorded | Not readable — Mech. ventilation (2) Start Time H H M M Not recorded | Not readable

Mech. ventilation (2) End Date Y Y Y Y M M D D Not recorded | Not readable — Mech. ventilation (2) End Time H H M M Not recorded | Not readable

Mechanical Ventilation #3

Oxygen saturation (%) level that led to the use of mechanical ventilation (3) [ ][ ][ ] % Not recorded | Not readable

Mech. ventilation (3) Start Date Y Y Y Y M M D D Not recorded | Not readable — Mech. ventilation (3) Start Time H H M M Not recorded | Not readable

Mech. ventilation (3) End Date Y Y Y Y M M D D Not recorded | Not readable — Mech. ventilation (3) End Time H H M M Not recorded | Not readable

Were antibiotics administered? Yes | No | Not recorded | Not readable

Tick if this antibiotic was administered — Was this antibiotic part of the initial treatment and/or part of a treatment change?

Antibiotic	Administered	Initial Treatment	Treatment Change	Discharge Treatment		
Benzathine Penicillin	☐	☐	☐	☐	Not recorded	Not readable
Crystalline Penicillin	☐	☐	☐	☐	Not recorded	Not readable
Gentamicin	☐	☐	☐	☐	Not recorded	Not readable
Amikacin	☐	☐	☐	☐	Not recorded	Not readable
Ceftriaxone	☐	☐	☐	☐	Not recorded	Not readable
Ceftazidime	☐	☐	☐	☐	Not recorded	Not readable
Cefotaxime	☐	☐	☐	☐	Not recorded	Not readable
Piperazine	☐	☐	☐	☐	Not recorded	Not readable
Metronidazole	☐	☐	☐	☐	Not recorded	Not readable
Vancomycin	☐	☐	☐	☐	Not recorded	Not readable
Clindamycin	☐	☐	☐	☐	Not recorded	Not readable
Ampicillin	☐	☐	☐	☐	Not recorded	Not readable
Amoxycillin	☐	☐	☐	☐	Not recorded	Not readable
Flucloxacillin	☐	☐	☐	☐	Not recorded	Not readable
Meropenem	☐	☐	☐	☐	Not recorded	Not readable
Levofloxacin	☐	☐	☐	☐	Not recorded	Not readable
Cloxacillin	☐	☐	☐	☐	Not recorded	Not readable

Neonatal Inpatient Form - Final Dataset

Abstractor Name [ ] Record ID [ ][ ][ ] - [ ][ ][ ][ ][ ] Date Y Y Y Y M M D D

Interventions (cont.)

Tick if this antibiotic was administered (cont.)

Was this antibiotic part of the initial treatment and/or part of a treatment change?

Antibiotic	Administered	Initial Treatment	Treatment Change	Discharge Treatment		
Other [ ]	☐	☐	☐	☐	Not recorded	Not readable
Other [ ]	☐	☐	☐	☐	Not recorded	Not readable
Other [ ]	☐	☐	☐	☐	Not recorded	Not readable

Treatment for Apnea: Yes / No / Not recorded / Not readable

If yes - Apnea Treatment Administered: Caffeine ☐ Aminophylline ☐ Not recorded / Not readable

Phototherapy Administered: Yes / No / Not recorded / Not readable

How many times did the newborn receive phototherapy? [ ][ ]

	Start Date		End Date	
Phototherapy (1)	Y Y Y Y M M D D	Not recorded / Not readable	Y Y Y Y M M D D	Not recorded / Not readable
Phototherapy (2)	Y Y Y Y M M D D	Not recorded / Not readable	Y Y Y Y M M D D	Not recorded / Not readable
Phototherapy (3)	Y Y Y Y M M D D	Not recorded / Not readable	Y Y Y Y M M D D	Not recorded / Not readable

IV fluids administered: Yes / No / Not recorded / Not readable

How many times were IV fluids administered? [ ][ ]

IV Fluids (First) Start Date: Y Y Y Y M M D D Not recorded / Not readable

IV Fluids (Last) End Date: Y Y Y Y M M D D Not recorded / Not readable

Transfusion administered: Yes - blood transfusion ☐ Yes - Exchange blood transfusion ☐ No ☐ Not recorded / Not readable

How many times was the infant given a blood transfusion or exchange blood transfusion? [ ][ ]

Transfusion (1) date: Y Y Y Y M M D D Not recorded / Not readable

Transfusion (2) date: Y Y Y Y M M D D Not recorded / Not readable

Transfusion (3) date: Y Y Y Y M M D D Not recorded / Not readable

Observations and Nursing Care

In what units was lowest recorded temperature measured?
°C ☐ °F ☐ Unknown / Not readable

In what units was highest recorded temperature measured?
°C ☐ °F ☐ Unknown / Not readable

Lowest recorded temperature [ ][ ].[ ] No / Not readable

Highest recorded temperature [ ][ ].[ ] No / Not readable

Lowest recorded oxygen saturation (%) [ ][ ] No / Not readable

Highest recorded oxygen saturation (%) [ ][ ] No / Not readable

Breast milk start date: Y Y Y Y M M D D Not recorded / Not readable

Feeding (other than breast milk) start date: Y Y Y Y M M D D Not recorded / Not readable

Feeding at discharge: Exclusive breastmilk ☐ Formula only ☐ Fortified breastmilk ☐ Predominant breastmilk ☐ Combination of breastmilk and formula ☐ Unknown - Absconder ☐ Not recorded / Not readable

In what units was lowest recorded blood sugar measured?
mmol/L ☐ mg/dL ☐ Unknown / Not readable

In what units was highest recorded blood sugar measured?
mmol/L ☐ mg/dL ☐ Unknown / Not readable

Lowest recorded blood sugar [ ][ ].[ ] No / Not readable

Highest recorded blood sugar [ ][ ].[ ] No / Not readable

Was bilirubin tested? Yes / No / Not recorded / Not readable

If yes - highest bilirubin value (mg/dL) [ ][ ].[ ] mg/dL No / Not readable

How was bilirubin measured? Serum ☐ Transcutaneous ☐ Not recorded / Not readable

Did the baby have seizures during admission? Yes / No / Not recorded / Not readable

Anticonvulsants administered: Yes / No / Not recorded / Not readable

Type of anticonvulsant: Phenobarbitone/Phenobarbitol ☐ Phenytoin ☐ Diazepam ☐ Not recorded / Not readable

Neonatal Inpatient Form - Final Dataset

NEST360°

Abstractor Name [ ] Record ID [ ][ ][ ] - [ ][ ][ ][ ][ ] Date Y Y Y Y M M D D

Discharge

Newborn status at discharge: Dead [ ] Alive [ ] Not recorded Not readable

Discharge Outcome
- Discharged alive [ ] Discharged with complications [ ] Discharged against medical advice - poor prognosis [ ]
- Discharged against medical advice - other [ ] Absconded [ ] Referred [ ] Not recorded Not readable

If referred - where? [ ]

Date of Discharge / Absconded / Death: Y Y Y Y M M D D Not recorded Not readable

Time of Discharge / Absconded / Death: H H M M Not recorded Not readable

Discharge Weight (grams) [ ][ ][ ][ ] g Not recorded Not readable

Was the patient given a follow-up appointment? Yes No Not recorded Not readable

If yes - appointment location: Neonatal Inpatient Unit (This Facility) [ ] Neonatal Outpatient Unit (This Facility) [ ] Other [ ]

Not recorded Not readable If other - where? [ ]

Please explain concisely why the patient WAS or WAS NOT given a follow-up appointment

[ ]

Final Discharge Diagnosis/Cause of Death (Primary #1)

Primary Category (1): A. Congenital Malformations [ ] B. Prematurity [ ] C. Infection [ ] D. Intrapartum-related [ ] E. Jaundice (Pathological) [ ] F. Other [ ] Not recorded Not readable

A. Sub-Categories of Congenital Malformations: Congenital Heart Defects [ ] Chromosomal Disorders [ ] Abdominal Defects [ ] Neural Tube Defects [ ] Orofacial Cleft [ ] Talipes/Club Foot [ ] Other Congenital [ ] Congenital Cause But Not Able to Specify [ ]

If other congenital cause/diagnosis - specify [ ]

B. Sub-Categories of Prematurity: Respiratory Distress Syndrome (of Prematurity) [ ] Intraventricular Haemorrhage [ ] Necrotizing Enterocolitis [ ] Other Prematurity [ ] Prematurity Cause But Not Able to Specify [ ]

If other prematurity cause/diagnosis - specify [ ]

C. Sub-Categories of Infection
- Probable Sepsis [ ] Culture-Positive Sepsis [ ] Probable Meningitis [ ] Culture-Positive Meningitis [ ]
- Pneumonia [ ] Tetanus [ ] SARS-CoV-2 (COVID-19) [ ] Other Infection [ ] Infection Cause But Not Able to Specify [ ]

If other infection cause/diagnosis - specify [ ]

Culture Positive Organism: Staphylococcus aureus [ ] Klebsiella spp. [ ] Pseudomonas spp. [ ] Escherichia coli [ ] Acinetobacter spp. [ ] Group B Streptococcus [ ] Other gram-negatives [ ] Other Streptococcus [ ] Other Fungal spp. [ ] Other [ ] Not indicated [ ] Not recorded Not readable

If other or spp. - full species/genus [ ]

Hospital-acquired infection? Yes No Not recorded Not readable

D. Sub-Categories of Intrapartum-Related: Neonatal Hypoxic Ischemic Encephalopathy [ ] Meconium Aspiration [ ] Birth Injury (Intrapartum-Related) [ ] Other Intrapartum-Related [ ] Intrapartum-Related Cause But Not Able to Specify [ ]

If other intrapartum-related cause/diagnosis - specify [ ]

E. Sub-Categories of Jaundice (Pathological): Requiring Phototherapy [ ] Requiring Exchange Transfusion [ ]

F. If Other Cause of Death or Final Diagnosis - specify [ ]

Final Discharge Diagnosis/Cause of Death (Primary #2)

Primary Category (2): A. Congenital Malformations [ ] B. Prematurity [ ] C. Infection [ ] D. Intrapartum-related [ ] E. Jaundice (Pathological) [ ] F. Other [ ] Not recorded Not readable

A. Sub-Categories of Congenital Malformations: Congenital Heart Defects [ ] Chromosomal Disorders [ ] Abdominal Defects [ ] Neural Tube Defects [ ] Orofacial Cleft [ ] Talipes/Club Foot [ ] Other Congenital [ ] Congenital Cause But Not Able to Specify [ ]

If other congenital cause/diagnosis - specify [ ]

Neonatal Inpatient Form - Final Dataset

Abstractor Name ☐ Record ID ☐☐☐ - ☐☐☐☐☐ Date YYYYMMDD

Discharge (cont.)

Final Discharge Diagnosis/Cause of Death (Primary #2) (cont.)

B. Sub-Categories of Prematurity: Respiratory Distress Syndrome (of Prematurity) ☐ Intraventricular Haemorrhage ☐ Necrotizing Enterocolitis ☐ Other Prematurity ☐ Prematurity Cause But Not Able to Specify ☐

If other prematurity cause/diagnosis - specify ☐

C. Sub-Categories of Infection

Probable Sepsis ☐ Culture-Positive Sepsis ☐ Probable Meningitis ☐ Culture-Positive Meningitis ☐
Pneumonia ☐ Tetanus ☐ SARS-CoV-2 (COVID-19) ☐ Other Infection ☐ Infection Cause But Not Able to Specify ☐

If other infection cause/diagnosis - specify ☐

Culture Positive Organism: Staphylococcus aureus ☐ Klebsiella spp. ☐ Pseudomonas spp. ☐
Escherichia coli ☐ Acinetobacter spp. ☐ Group B Streptococcus ☐ Other gram-negatives ☐
Other Streptococcus ☐ Other Fungal spp. ☐ Other ☐ Not indicated ☐ Not recorded Not readable

If other or spp. - full species/genus ☐

Hospital-acquired infection? Yes No Not recorded Not readable

D. Sub-Categories of Intrapartum-Related

Neonatal Hypoxic Ischemic Encephalopathy ☐ Meconium Aspiration ☐ Birth Injury (Intrapartum-Related) ☐
Other Intrapartum-Related ☐ Intrapartum-Related Cause But Not Able to Specify ☐

If other intrapartum-related cause/diagnosis - specify ☐

E. Sub-Categories of Jaundice (Pathological): Requiring Phototherapy ☐ Requiring Exchange Transfusion ☐

F. If Other Cause of Death or Final Diagnosis - specify ☐

Final Discharge Diagnosis/Cause of Death (Primary #3)

Primary Category (3): A. Congenital Malformations ☐ B. Prematurity ☐ C. Infection ☐
D. Intrapartum-related ☐ E. Jaundice (Pathological) ☐ F. Other ☐ Not recorded Not readable

A. Sub-Categories of Congenital Malformations: Congenital Heart Defects ☐ Chromosomal Disorders ☐ Abdominal Defects ☐
Neural Tube Defects ☐ Orofacial Cleft ☐ Talipes/Club Foot ☐ Other Congenital ☐ Congenital Cause But Not Able to Specify ☐

If other congenital cause/diagnosis - specify ☐

B. Sub-Categories of Prematurity: Respiratory Distress Syndrome (of Prematurity) ☐ Intraventricular Haemorrhage ☐ Necrotizing Enterocolitis ☐ Other Prematurity ☐ Prematurity Cause But Not Able to Specify ☐

If other prematurity cause/diagnosis - specify ☐

C. Sub-Categories of Infection

Probable Sepsis ☐ Culture-Positive Sepsis ☐ Probable Meningitis ☐ Culture-Positive Meningitis ☐
Pneumonia ☐ Tetanus ☐ SARS-CoV-2 (COVID-19) ☐ Other Infection ☐ Infection Cause But Not Able to Specify ☐

If other infection cause/diagnosis - specify ☐

Culture Positive Organism: Staphylococcus aureus ☐ Klebsiella spp. ☐ Pseudomonas spp. ☐
Escherichia coli ☐ Acinetobacter spp. ☐ Group B Streptococcus ☐ Other gram-negatives ☐
Other Streptococcus ☐ Other Fungal spp. ☐ Other ☐ Not indicated ☐ Not recorded Not readable

If other or spp. - full species/genus ☐

Hospital-acquired infection? Yes No Not recorded Not readable

D. Sub-Categories of Intrapartum-Related

Neonatal Hypoxic Ischemic Encephalopathy ☐ Meconium Aspiration ☐ Birth Injury (Intrapartum-Related) ☐
Other Intrapartum-Related ☐ Intrapartum-Related Cause But Not Able to Specify ☐

If other intrapartum-related cause/diagnosis - specify ☐

E. Sub-Categories of Jaundice (Pathological): Requiring Phototherapy ☐ Requiring Exchange Transfusion ☐

F. If Other Cause of Death or Final Diagnosis - specify ☐

Neonatal Inpatient Form - Final Dataset

Abstractor Name ☐ Record ID ☐☐☐ - ☐☐☐☐☐ Date Y Y Y Y M M D D

Discharge (cont.)

Final Discharge Diagnosis/Cause of Death (Secondary #1)

Secondary Category (1) A. Congenital Malformations ☐ B. Prematurity ☐ C. Infection ☐
D. Intrapartum-related ☐ E. Jaundice (Pathological) ☐ F. Other ☐ Not recorded Not readable

A. Sub-Categories of Congenital Malformations Congenital Heart Defects ☐ Chromosomal Disorders ☐ Abdominal Defects ☐
Neural Tube Defects ☐ Orofacial Cleft ☐ Talipes/Club Foot ☐ Other Congenital ☐ Congenital Cause But Not Able to Specify ☐

If other congenital cause/diagnosis - specify ☐

B. Sub-Categories of Prematurity Respiratory Distress Syndrome (of Prematurity) ☐ Intraventricular Haemorrhage ☐
Necrotizing Enterocolitis ☐ Other Prematurity ☐ Prematurity Cause But Not Able to Specify ☐

If other prematurity cause/diagnosis - specify ☐

C. Sub-Categories of Infection
Probable Sepsis ☐ Culture-Positive Sepsis ☐ Probable Meningitis ☐ Culture-Positive Meningitis ☐
Pneumonia ☐ Tetanus ☐ SARS-CoV-2 (COVID-19) ☐ Other Infection ☐ Infection Cause But Not Able to Specify ☐

If other infection cause/diagnosis - specify ☐

Culture Positive Organism Staphylococcus aureus ☐ Klebsiella spp. ☐ Pseudomonas spp. ☐
Escherichia coli ☐ Acinetobacter spp. ☐ Group B Streptococcus ☐ Other gram-negatives ☐
Other Streptococcus ☐ Other Fungal spp. ☐ Other ☐ Not indicated ☐ Not recorded Not readable

If other or spp. - full species/genus ☐

Hospital-acquired infection? Yes No Not recorded Not readable

D. Sub-Categories of Intrapartum-Related
Neonatal Hypoxic Ischemic Encephalopathy ☐ Meconium Aspiration ☐ Birth Injury (Intrapartum-Related) ☐
Other Intrapartum-Related ☐ Intrapartum-Related Cause But Not Able to Specify ☐

If other intrapartum-related cause/diagnosis - specify ☐

E. Sub-Categories of Jaundice (Pathological) Requiring Phototherapy ☐ Requiring Exchange Transfusion ☐

F. If Other Cause of Death or Final Diagnosis - specify ☐

Final Discharge Diagnosis/Cause of Death (Secondary #2)

Secondary Category (2) A. Congenital Malformations ☐ B. Prematurity ☐ C. Infection ☐
D. Intrapartum-related ☐ E. Jaundice (Pathological) ☐ F. Other ☐ Not recorded Not readable

A. Sub-Categories of Congenital Malformations Congenital Heart Defects ☐ Chromosomal Disorders ☐ Abdominal Defects ☐
Neural Tube Defects ☐ Orofacial Cleft ☐ Talipes/Club Foot ☐ Other Congenital ☐ Congenital Cause But Not Able to Specify ☐

If other congenital cause/diagnosis - specify ☐

B. Sub-Categories of Prematurity Respiratory Distress Syndrome (of Prematurity) ☐ Intraventricular Haemorrhage ☐
Necrotizing Enterocolitis ☐ Other Prematurity ☐ Prematurity Cause But Not Able to Specify ☐

If other prematurity cause/diagnosis - specify ☐

C. Sub-Categories of Infection
Probable Sepsis ☐ Culture-Positive Sepsis ☐ Probable Meningitis ☐ Culture-Positive Meningitis ☐
Pneumonia ☐ Tetanus ☐ SARS-CoV-2 (COVID-19) ☐ Other Infection ☐ Infection Cause But Not Able to Specify ☐

If other infection cause/diagnosis - specify ☐

Culture Positive Organism Staphylococcus aureus ☐ Klebsiella spp. ☐ Pseudomonas spp. ☐
Escherichia coli ☐ Acinetobacter spp. ☐ Group B Streptococcus ☐ Other gram-negatives ☐
Other Streptococcus ☐ Other Fungal spp. ☐ Other ☐ Not indicated ☐ Not recorded Not readable

If other or spp. - full species/genus ☐

Hospital-acquired infection? Yes No Not recorded Not readable

Neonatal Inpatient Form - Final Dataset

Abstractor Name ☐ Record ID ☐☐☐ - ☐☐☐☐☐ Date Y Y Y Y M M D D

Discharge (cont.)

Final Discharge Diagnosis/Cause of Death (Secondary #2) (cont.)

D. Sub-Categories of Intrapartum-Related

Neonatal Hypoxic Ischemic Encephalopathy ☐ Meconium Aspiration ☐ Birth Injury (Intrapartum-Related) ☐
Other Intrapartum-Related ☐ Intrapartum-Related Cause But Not Able to Specify ☐

If other intrapartum-related cause/diagnosis - specify ☐

E. Sub-Categories of Jaundice (Pathological) Requiring Phototherapy ☐ Requiring Exchange Transfusion ☐

F. If Other Cause of Death or Final Diagnosis - specify ☐

Final Discharge Diagnosis/Cause of Death (Secondary #3)

Secondary Category (3) A. Congenital Malformations ☐ B. Prematurity ☐ C. Infection ☐
D. Intrapartum-related ☐ E. Jaundice (Pathological) ☐ F. Other ☐ Not recorded Not readable

A. Sub-Categories of Congenital Malformations Congenital Heart Defects ☐ Chromosomal Disorders ☐ Abdominal Defects ☐
Neural Tube Defects ☐ Orofacial Cleft ☐ Talipes/Club Foot ☐ Other Congenital ☐ Congenital Cause But Not Able to Specify ☐

If other congenital cause/diagnosis - specify ☐

B. Sub-Categories of Prematurity Respiratory Distress Syndrome (of Prematurity) ☐ Intraventricular Haemorrhage ☐
Necrotizing Enterocolitis ☐ Other Prematurity ☐ Prematurity Cause But Not Able to Specify ☐

If other prematurity cause/diagnosis - specify ☐

C. Sub-Categories of Infection

Probable Sepsis ☐ Culture-Positive Sepsis ☐ Probable Meningitis ☐ Culture-Positive Meningitis ☐
Pneumonia ☐ Tetanus ☐ SARS-CoV-2 (COVID-19) ☐ Other Infection ☐ Infection Cause But Not Able to Specify ☐

If other prematurity cause/diagnosis - specify ☐

Culture Positive Organism Staphylococcus aureus ☐ Klebsiella spp. ☐ Pseudomonas spp. ☐
Escherichia coli ☐ Acinetobacter spp. ☐ Group B Streptococcus ☐ Other gram-negatives ☐
Other Streptococcus ☐ Other Fungal spp. ☐ Other ☐ Not indicated ☐ Not recorded Not readable

If other or spp. - full species/genus ☐

Hospital-acquired infection? Yes No Not recorded Not readable

D. Sub-Categories of Intrapartum-Related

Neonatal Hypoxic Ischemic Encephalopathy ☐ Meconium Aspiration ☐ Birth Injury (Intrapartum-Related) ☐
Other Intrapartum-Related ☐ Intrapartum-Related Cause But Not Able to Specify ☐

If other intrapartum-related cause/diagnosis - specify ☐

E. Sub-Categories of Jaundice (Pathological) Requiring Phototherapy ☐ Requiring Exchange Transfusion ☐

F. If Other Cause of Death or Final Diagnosis - specify ☐

Was LBW mentioned as a diagnosis or cause of death? Yes ☐ No ☐

Additional comments

☐

END OF FORM
