# Supplementary material for: Neonatal inpatient dataset for small and sick newborn care in low- and middle-income countries: systematic development and multi-country operationalisation with NEST360
Source: BMC Pediatr. 2023 Nov 15;23(Suppl 2):567. doi: 10.1186/s12887-023-04341-2 (PMC10652643; doi:10.1186/s12887-023-04341-2)
Supplement: Supplementary file 4 — Additional file 4. Neonatal Inpatient Dataset data flow. [file 12887_2023_4341_MOESM4_ESM.pdf]

SUPPLEMENTAL INFORMATION – ADDITIONAL FILE 4

SUPPLEMENT TITLE

Small and sick newborn care: African-led implementation research

PAPER TITLE

Draft for Discussion

Neonatal inpatient dataset for small and sick newborn care in low- and middle-income countries: systematic development and multi-country operationalisation with NEST360.

Additional File 4: *Neonatal Inpatient Dataset data flow*

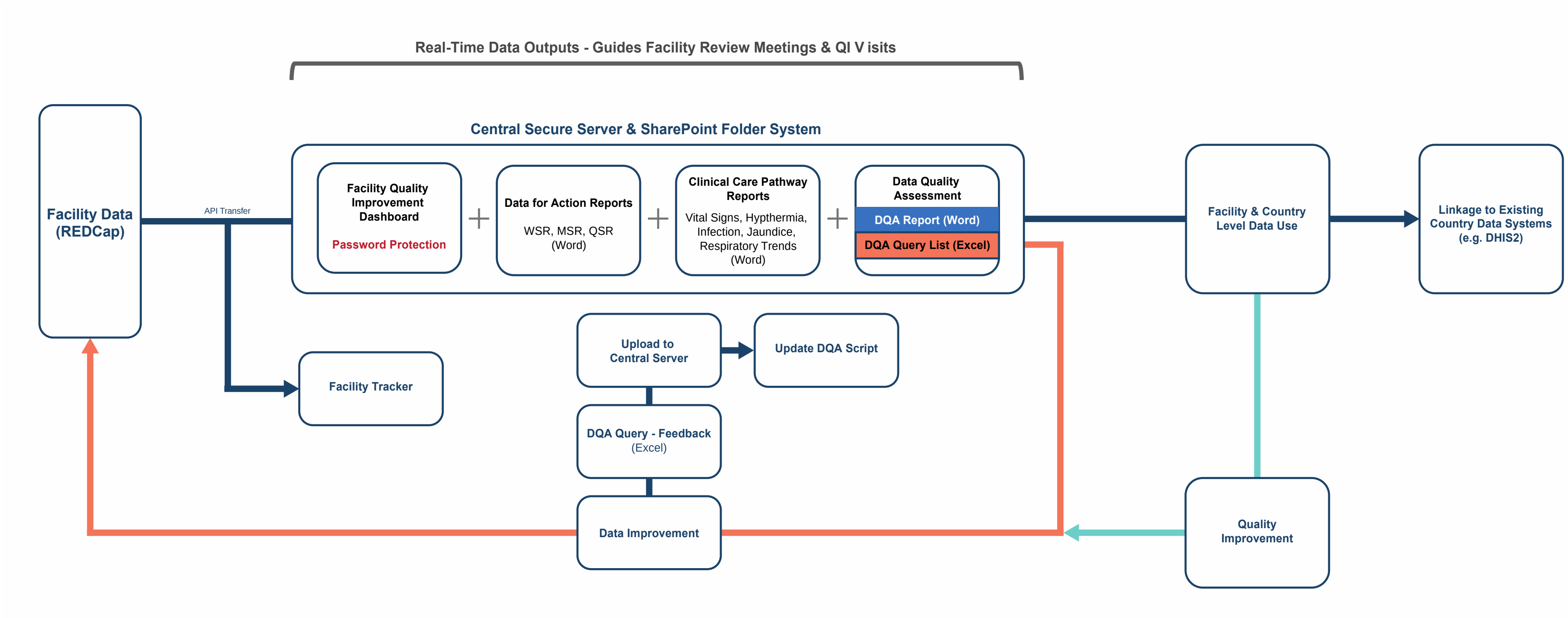

The data flow diagram represents data management from a REDCap server to a central server. Pooled data are aggregated to form country and facility-level quality improvement dashboards. Data is also used to formulate data for action, clinical care pathway and data quality assessment reports and query lists. Data quality assessment query lists inform data improvement by country team manual data edits and feedback. Edited query lists are uploaded into the central server to update the next data quality assessment. All reports and the dashboard support care quality improvement at review meetings and QI visits. Individual-level data links to existing country and facility data systems.

Legend: Excel is a spreadsheet software developed by Microsoft. SharePoint is a web-based collaborative platform developed by Microsoft. Word is a word-processing software developed by Microsoft. The shiny package in R software generates quality improvement facility dashboards. Data for action, clinical care pathways, data quality assessment reports and query lists are automated using R software. Data management scripts are produced using STATA and R Software.

Abbreviations: WSR, Weekly Summary Report; MSR, Monthly Summary Report; QSR, Quarterly Summary Report; QI, Quality Improvement; DQA, Data Quality Assessment; API, Application Programming Interface; N, number; REDCap, Research Electronic Data Capture.

Draft for Discussion
