# Supplementary material for: Neonatal inpatient dataset for small and sick newborn care in low- and middle-income countries: systematic development and multi-country operationalisation with NEST360
Source: BMC Pediatr. 2023 Nov 15;23(Suppl 2):567. doi: 10.1186/s12887-023-04341-2 (PMC10652643; doi:10.1186/s12887-023-04341-2)
Supplement: Supplementary file 7 — Additional file 7. Evaluating facility-level data entry timeliness in Malawi, Kenya, Tanzania, and Nigeria. [file 12887_2023_4341_MOESM7_ESM.pdf]

# SUPPLEMENTAL INFORMATION – ADDITIONAL FILE 7

## SUPPLEMENT TITLE

**Small and sick newborn care: African-led implementation research**

## PAPER TITLE

Draft for Discussion

**Neonatal inpatient dataset for small and sick newborn care in low- and middle-income countries: systematic development and multi-country operationalisation with NEST360.**

Additional File 7: *Evaluating facility-level data entry timeliness in Malawi, Kenya, Tanzania, and Nigeria.*

**Figure 1: Evaluating the impact of onsite facility-level data entry on data timeliness in Malawi: a comparison of the percentage of total admission records with delayed data entry (colour-coded) pre- (A) and post- (B) operationalisation of onsite data collectors.**

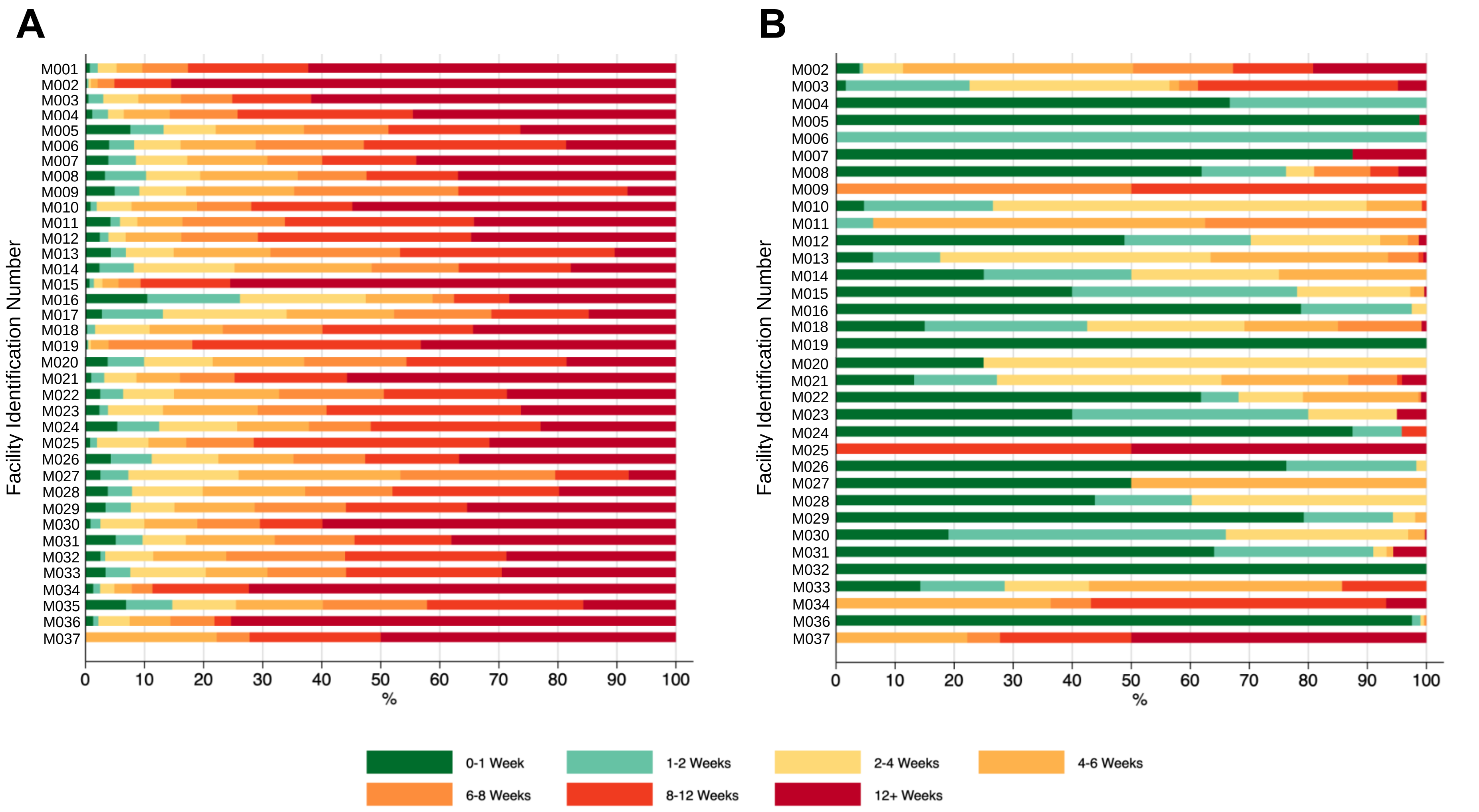

**Legend**: Analysis of time delay from newborn discharge to form data entry into REDCap database for each admission by facility. Records entered using off-site data entry (A) comparing to records entered on-site via desktop computer (B).

Draft for Discussion

**Figure 2: Evaluating the impact of onsite facility-level data entry on data timeliness in Kenya, Tanzania, and Nigeria: the percentage of total admission records with delayed data entry (colour-coded).**

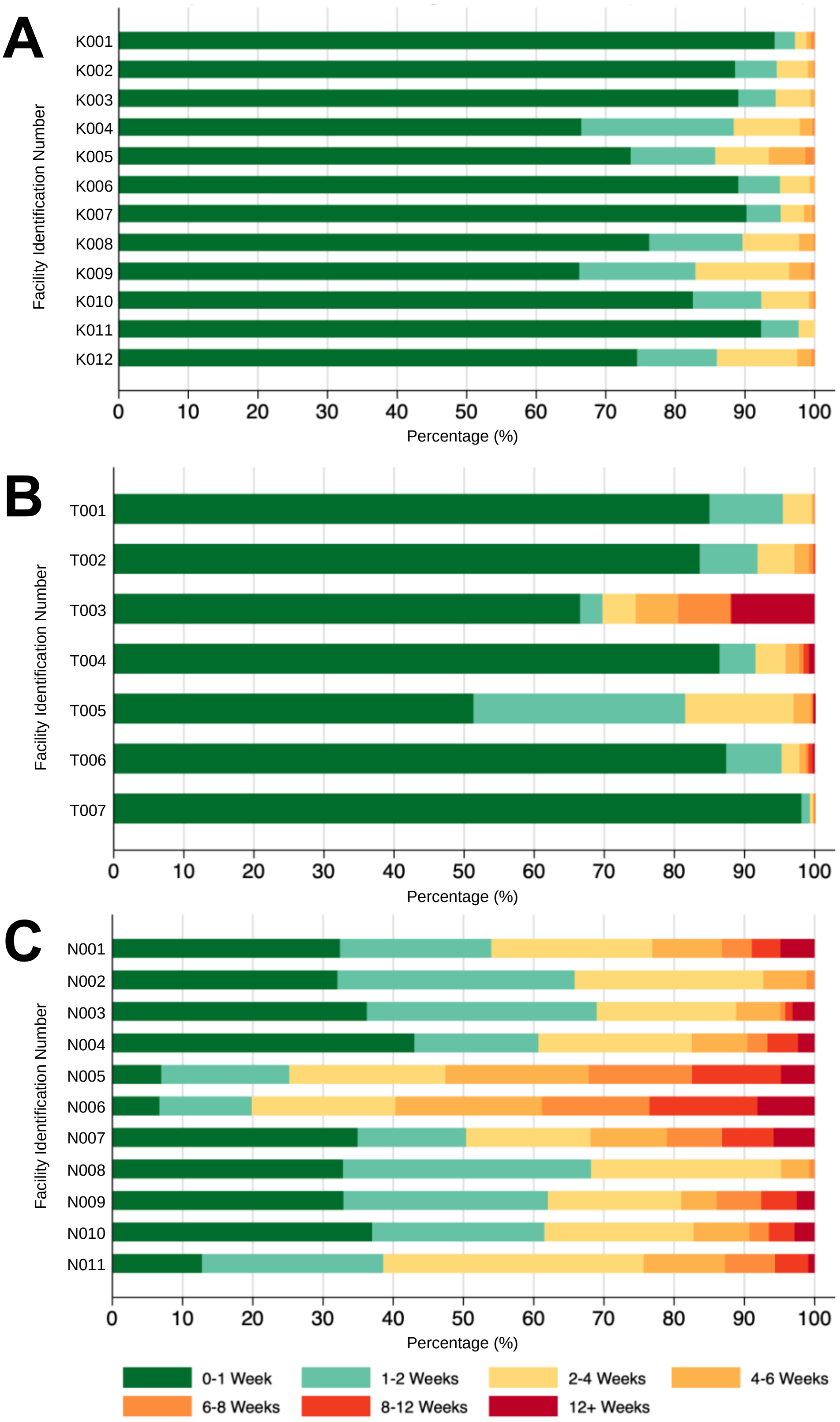

**Legend**: Analysis of time delay from newborn discharge to form data entry into REDCap database for each admission by facility. Records entered in Kenya (A, on-site and electronic data entry), Tanzania (B, on-site and electronic data entry) and Nigeria (C, on-site and paper data entry) from 1st January 2021 to 1st January 2023.

Draft for Discussion
