# Supplementary material for: Neonatal inpatient dataset for small and sick newborn care in low- and middle-income countries: systematic development and multi-country operationalisation with NEST360
Source: BMC Pediatr. 2023 Nov 15;23(Suppl 2):567. doi: 10.1186/s12887-023-04341-2 (PMC10652643; doi:10.1186/s12887-023-04341-2)
Supplement: Supplementary file 8 — Additional file 8. NEST360 Neonatal Inpatient Dataset data collection infrastructure by facility. [file 12887_2023_4341_MOESM8_ESM.pdf]

SUPPLEMENTAL INFORMATION – ADDITIONAL FILE 8

SUPPLEMENT TITLE

Small and sick newborn care: African-led implementation research

PAPER TITLE

Neonatal inpatient dataset for small and sick newborn care in low- and middle-income countries: systematic development and multi-country operationalisation with NEST360.

Additional File 8: *NEST360 Neonatal Inpatient Dataset data collection infrastructure by facility.*

Facility ID	Country	Number of Data Collectors (On-Site)	Data Collector Employer	Internet Access (On-Site)	Internet Provider	Device Used	Device Provider
M001	Malawi	1	Malawi Ministry of Health	Yes	Malawi Ministry of Health	Desktop Computer	NEST360
M002	Malawi	1	Malawi Ministry of Health	Yes	Malawi Ministry of Health	Desktop Computer	NEST360
M003	Malawi	1	Malawi Ministry of Health	Yes	Malawi Ministry of Health	Desktop Computer	NEST360

M004	Malawi	1	Malawi Ministry of Health	Yes	Malawi Ministry of Health	Desktop Computer	NEST360
M005	Malawi	1	Malawi Ministry of Health	Yes	Malawi Ministry of Health	Desktop Computer	NEST360
M006	Malawi	1	Malawi Ministry of Health	Yes	Malawi Ministry of Health	Desktop Computer	NEST360
M007	Malawi	1	Malawi Ministry of Health	Yes	Malawi Ministry of Health	Desktop Computer	NEST360
M008	Malawi	1	Malawi Ministry of Health	Yes	Malawi Ministry of Health	Desktop Computer	NEST360
M009	Malawi	1	Malawi Ministry of Health	Yes	Malawi Ministry of Health	Desktop Computer	NEST360
M010	Malawi	1	Malawi Ministry of Health	Yes	Malawi Ministry of Health	Desktop Computer	NEST360
M011	Malawi	1	Malawi Ministry of Health	Yes	Malawi Ministry of Health	Desktop Computer	NEST360
M012	Malawi	1	Malawi Ministry of Health	Yes	Malawi Ministry of Health	Desktop Computer	NEST360
M013	Malawi	1	Malawi Ministry of Health	Yes	Malawi Ministry of Health	Desktop Computer	NEST360
M014	Malawi	1	Malawi Ministry of Health	Yes	Malawi Ministry of Health	Desktop Computer	NEST360
M015	Malawi	1	Malawi Ministry of Health	Yes	Malawi Ministry of Health	Desktop Computer	NEST360
M016	Malawi	1	Malawi Ministry of Health	Yes	Malawi Ministry of Health	Desktop Computer	NEST360
M017	Malawi	1	Malawi Ministry of Health	Yes	Malawi Ministry of Health	Desktop Computer	NEST360
M018	Malawi	1	Malawi Ministry of Health	Yes	Malawi Ministry of Health	Desktop Computer	NEST360
M019	Malawi	1	Malawi Ministry of Health	Yes	Malawi Ministry of Health	Desktop Computer	NEST360
M020	Malawi	1	Malawi Ministry of Health	Yes	Malawi Ministry of Health	Desktop Computer	NEST360
M021	Malawi	1	Malawi Ministry of Health	Yes	Malawi Ministry of Health	Desktop Computer	NEST360
M022	Malawi	1	Malawi Ministry of Health	Yes	Malawi Ministry of Health	Desktop Computer	NEST360
M023	Malawi	1	Malawi Ministry of Health	Yes	Malawi Ministry of Health	Desktop Computer	NEST360
M024	Malawi	1	Malawi Ministry of Health	Yes	Malawi Ministry of Health	Desktop Computer	NEST360
M025	Malawi	1	Malawi Ministry of Health	Yes	Malawi Ministry of Health	Desktop Computer	NEST360
M026	Malawi	1	Malawi Ministry of Health	Yes	Malawi Ministry of Health	Desktop Computer	NEST360
M027	Malawi	1	Malawi Ministry of Health	Yes	Malawi Ministry of Health	Desktop Computer	NEST360
M028	Malawi	1	Malawi Ministry of Health	Yes	Malawi Ministry of Health	Desktop Computer	NEST360
M029	Malawi	1	Malawi Ministry of Health	Yes	Malawi Ministry of Health	Desktop Computer	NEST360
M030	Malawi	1	Malawi Ministry of Health	Yes	Malawi Ministry of Health	Desktop Computer	NEST360
M031	Malawi	1	Malawi Ministry of Health	Yes	Malawi Ministry of Health	Desktop Computer	NEST360

M032	Malawi	1	Malawi Ministry of Health	Yes	Malawi Ministry of Health	Desktop Computer	NEST360
M033	Malawi	1	Malawi Ministry of Health	Yes	Malawi Ministry of Health	Desktop Computer	NEST360
M034	Malawi	1	Malawi Ministry of Health	Yes	Malawi Ministry of Health	Desktop Computer	NEST360
M035	Malawi	1	Malawi Ministry of Health	Yes	Malawi Ministry of Health	Desktop Computer	NEST360
M036	Malawi	1	Malawi Ministry of Health	Yes	Malawi Ministry of Health	Desktop Computer	NEST360
M037	Malawi	1	Malawi Ministry of Health	Yes	Malawi Ministry of Health	Desktop Computer	NEST360
M038	Malawi	1	Malawi Ministry of Health	Yes	Malawi Ministry of Health	Desktop Computer	NEST360
K001	Kenya	1	KEPRECON (KEMRI-Wellcome Trust Grant)	Yes	CIN (KEMRI-Wellcome Trust Grant)	Desktop Computer	CIN (KEMRI-Wellcome Trust Grant)
K002	Kenya	1	KEPRECON (KEMRI-Wellcome Trust Grant)	Yes	CIN (KEMRI-Wellcome Trust Grant)	Desktop Computer	CIN (KEMRI-Wellcome Trust Grant)
K003	Kenya	1	KEPRECON (KEMRI-Wellcome Trust Grant)	Yes	CIN (KEMRI-Wellcome Trust Grant)	Desktop Computer	CIN (KEMRI-Wellcome Trust Grant)
K004	Kenya	1	KEPRECON (KEMRI-Wellcome Trust Grant)	Yes	CIN (KEMRI-Wellcome Trust Grant)	Desktop Computer	CIN (KEMRI-Wellcome Trust Grant)
K005	Kenya	1	KEPRECON (KEMRI-Wellcome Trust Grant)	Yes	CIN (KEMRI-Wellcome Trust Grant)	Desktop Computer	CIN (KEMRI-Wellcome Trust Grant)
K006	Kenya	1	KEPRECON (KEMRI-Wellcome Trust Grant)	Yes	CIN (KEMRI-Wellcome Trust Grant)	Desktop Computer	CIN (KEMRI-Wellcome Trust Grant)
K007	Kenya	1	KEPRECON (KEMRI-Wellcome Trust Grant)	Yes	CIN (KEMRI-Wellcome Trust Grant)	Desktop Computer	CIN (KEMRI-Wellcome Trust Grant)
K008	Kenya	1	KEPRECON (KEMRI-Wellcome Trust Grant)	Yes	CIN (KEMRI-Wellcome Trust Grant)	Desktop Computer	CIN (KEMRI-Wellcome Trust Grant)
K009	Kenya	1	KEPRECON (KEMRI-Wellcome Trust Grant)	Yes	CIN (KEMRI-Wellcome Trust Grant)	Desktop Computer	CIN (KEMRI-Wellcome Trust Grant)
K010	Kenya	1	KEPRECON (KEMRI-Wellcome Trust Grant)	Yes	CIN (KEMRI-Wellcome Trust Grant)	Desktop Computer	CIN (KEMRI-Wellcome Trust Grant)
K011	Kenya	1	KEPRECON (KEMRI-Wellcome Trust Grant)	Yes	CIN (KEMRI-Wellcome Trust Grant)	Desktop Computer	CIN (KEMRI-Wellcome Trust Grant)
K012	Kenya	2	KEPRECON (KEMRI-Wellcome Trust Grant)	Yes	CIN (KEMRI-Wellcome Trust Grant)	Desktop Computer	CIN (KEMRI-Wellcome Trust Grant)
K013	Kenya	1	KEPRECON (KEMRI-Wellcome Trust Grant)	Yes	CIN (KEMRI-Wellcome Trust Grant)	Desktop Computer	CIN (KEMRI-Wellcome Trust Grant)
T001	Tanzania	2	NEST360/IHI	Yes	NEST360/IHI	Tablet & Laptop Computer	NEST360/IHI
T002	Tanzania	2	NEST360/IHI	Yes	NEST360/IHI	Tablet & Laptop Computer	NEST360/IHI
T003	Tanzania	2	NEST360/IHI	Yes	NEST360/IHI	Laptop Computer	NEST360/IHI
T004	Tanzania	2	NEST360/IHI	Yes	NEST360/IHI	Tablet	NEST360/IHI
T005	Tanzania	2	NEST360/IHI	Yes	NEST360/IHI	Tablet & Laptop Computer	NEST360/IHI
T006	Tanzania	2	NEST360/IHI	Yes	NEST360/IHI	Tablet & Laptop Computer	NEST360/IHI

T007	Tanzania	2	NEST360/IHI	Yes	NEST360/IHI	Tablet	NEST360/IHI
N001	Nigeria	0.5	NEST360/APIN	Yes	NEST360/APIN	Laptop Computer	NEST360/APIN
N002	Nigeria	0.5	NEST360/APIN	Yes	NEST360/APIN	Laptop Computer	NEST360/APIN
N003	Nigeria	0.25	NEST360/APIN	Yes	NEST360/APIN	Laptop Computer	NEST360/APIN
N004	Nigeria	0.5	NEST360/APIN	Yes	NEST360/APIN	Laptop Computer	NEST360/APIN
N005	Nigeria	0.5	NEST360/APIN	Yes	NEST360/APIN	Laptop Computer	NEST360/APIN
N006	Nigeria	0.5	NEST360/APIN	Yes	NEST360/APIN	Laptop Computer	NEST360/APIN
N007	Nigeria	0.5	NEST360/APIN	Yes	NEST360/APIN	Laptop Computer	NEST360/APIN
N008	Nigeria	0.5	NEST360/APIN	Yes	NEST360/APIN	Laptop Computer	NEST360/APIN
N009	Nigeria	0.25	NEST360/APIN	Yes	NEST360/APIN	Laptop Computer	NEST360/APIN
N010	Nigeria	0.5	NEST360/APIN	Yes	NEST360/APIN	Laptop Computer	NEST360/APIN
N011	Nigeria	0.5	NEST360/APIN	Yes	NEST360/APIN	Laptop Computer	NEST360/APIN

Abbreviations: ID, Identity;, IHI, Ifakara Health Institute; KEMRI, Kenya Medical Research Institute; APIN, AIDS Prevention Initiative in Nigeria; CIN, Clinical Information Network; KEPRECON, Kenya Paediatric Research Consortium.

Draft for Discussion
