# Supplementary material for: Neonatal inpatient dataset for small and sick newborn care in low- and middle-income countries: systematic development and multi-country operationalisation with NEST360
Source: BMC Pediatr. 2023 Nov 15;23(Suppl 2):567. doi: 10.1186/s12887-023-04341-2 (PMC10652643; doi:10.1186/s12887-023-04341-2)
Supplement: Supplementary file 9 — Additional file 9. Local ethical approval for the complex evaluation of the implementation of a small and sick newborn care package with NEST360. [file 12887_2023_4341_MOESM9_ESM.pdf]

SUPPLEMENTAL INFORMATION – ADDITIONAL FILE 9

SUPPLEMENT TITLE

Small and sick newborn care: African-led implementation research

PAPER TITLE

Neonatal inpatient dataset for small and sick newborn care in low- and middle-income countries: systematic development and multi-country operationalisation with NEST360.

Additional File 9: *Local ethical approval for the complex evaluation of the implementation of a small and sick newborn care package with NEST360*

Country	Protocol Title	LEC Protocol ID
**Kenya**	Using a Health Facility Assessment to Assess Quality of Newborn Care in Kenya	MSU/DRPI/MUERC/00810/19
**Malawi**	Using a Health Facility Assessment to Assess Quality of Newborn Care in Malawi	NHSRC 2463
**Nigeria**	Quality Improvement Study of the Implementation of a Package of Trainings and Technologies for the Delivery of Comprehensive Newborn Care in Nigeria: A Multi-Country Study	**LUTH:** ADM/DCST/HREC/APP/3487<br>**UCH:** UI/EC/20/0713<br>**NHREC:** NHREC/01/01/2007
**Tanzania**		**IHI:** IHI/IRB/01-2021

	Implementation study to improve the quality of comprehensive newborn care through introduction of the package of Newborn Essential Solutions and Technologies (NEST) in Tanzania	**MUHAS:** MUHAS-REC-12-2019-072<br><br>**NIMR:** 3405

Abbreviations: LEC; Local Ethics Committee, ID; Identity, MSU; Michigan State University, DRPI; Disability Right Promotion International, MUERC; Maseno University Ethics Review Committee, NHSRC; National Health Science Research Committee, LUTH; Lagos University Teaching Hospital, UCH; University College Hospital, NHREC; National Health Research Ethics Committee, IHI; Ifakara Health Institute, MUHAS; Muhimbili University of Health and Allied Science, NIMR; National Institute for Medical Research

1

Draft for Discussion
